# Prediction of aflatoxin contamination outbreaks in Texas corn using mechanistic and machine learning models

**DOI:** 10.3389/fmicb.2025.1528997

**Published:** 2025-03-05

**Authors:** Lina Castano-Duque, Angela Avila, Brian M. Mack, H. Edwin Winzeler, Joshua M. Blackstock, Matthew D. Lebar, Geromy G. Moore, Phillip Ray Owens, Hillary L. Mehl, Jianzhong Su, James Lindsay, Kanniah Rajasekaran

**Affiliations:** ^1^USDA, Agriculture Research Service, Southern Regional Research Center, New Orleans, LA, United States; ^2^Department of Mathematics, University of Texas, Arlington, TX, United States; ^3^USDA, Agriculture Research Service, Dale Bumpers Small Farms Research Center, Booneville, AR, United States; ^4^Center for Advanced Spatial Technologies, University of Arkansas, Fayetteville, AR, United States; ^5^Department of Geosciences, University of Arkansas, Fayetteville, AR, United States; ^6^USDA, Agriculture Research Service, Arid Land Agricultural Research Center, Tucson, AZ, United States; ^7^Office of National Programs, Agriculture Research Service, USDA, Beltsville, MD, United States

**Keywords:** *Aspergillus*, machine learning, gradient boosting, neural network, aflatoxin, soil, corn

## Abstract

Aflatoxins are carcinogenic and mutagenic mycotoxins that contaminate food and feed. The objective of our research is to predict aflatoxin outbreaks in Texas-grown maize using dynamic geospatial data from remote sensing satellites, soil properties data, and meteorological data by an ensemble of models. We developed three model pipelines: two included mechanistic models that use weekly aflatoxin risk indexes (ARIs) as inputs, and one included a weather-centric model; all three models incorporated soil properties as inputs. For the mechanistic-dependent models, ARIs were weighted based on a maize phenological model that used satellite-acquired normalized difference vegetation index (NDVI) data to predict maize planting dates for each growing season on a county basis. For aflatoxin outbreak predictions, we trained, tested and validated gradient boosting and neural network models using inputs of ARIs or weather, soil properties, and county geodynamic latitude and longitude references. Our findings indicated that between the two ARI-mechanistic models evaluated (AFLA-MAIZE or Ratkowsky), the best performing was the Ratkowsky-ARI neural network (nnet) model, with an accuracy of 73%, sensitivity of 71% and specificity of 74%. Texas has significant geographical variability in ARI and ARI-hotspot responses due to the diversity of agroecological zones (hot-dry, hot-humid, mixed-dry and mixed-humid) that result in a wide variation of maize growth and development. Our Ratkowsky-ARI nnet model identified a positive correlation between aflatoxin outbreaks and prevalence of ARI hot-spots in the hot-humid areas of Texas. In these areas, temperature, precipitation and relative humidity in March and October were positively correlated with high aflatoxin contamination events. We found a positive correlation between aflatoxin outbreaks and soil pH in hot-dry and hot-humid regions and minimum saturated hydraulic conductivity in mixed-dry regions. Conversely, there was a negative relationship between aflatoxin outbreaks and maximum soil organic matter (hot-dry region), and calcium carbonate (hot-dry, and mixed-dry). It is likely soil fungal communities are more diverse, and plants are healthier in soils with high organic matter content, thereby reducing the risk of aflatoxin outbreaks. Our results demonstrate that intricate relationships between soil hydrological parameters, fungal communities and plant health should be carefully considered by Texas corn growers for aflatoxin mitigation strategies.

## 1 Introduction

Aflatoxins (AFLs) are toxic secondary metabolites produced by some species of *Aspergillus* and are a major safety and seed quality concern worldwide (Munkvold et al., 2019; Wu et al., 2014). AFLs not only pose health risks to humans and other animals through aflatoxicosis and carcinogenesis, but they also cause substantial economic loss (Mitchell et al., 2016). Contaminated maize kernels with AFL concentrations exceeding U.S. Food and Drug Administration (FDA) action levels (20 ppb for human consumption) must be either discarded or used for other purposes, thereby reducing market value of the crop (Mitchell et al., 2016; Vardon et al., 2003). Multiple sectors of the agricultural industry (growers, processors, and consumers) are negatively affected by mycotoxin contamination of grain, with billions of dollars in annual losses (Vardon et al., 2003; Wu, 2006; Mitchell et al., 2016). *Aspergillus flavus* is a well-known producer of AFL that can grow and produce mycotoxins at a wide range of temperatures, with optimal growth between 30 and 35°C (Abdel-Hadi et al., 2012). Dry, hot conditions favor *A. flavus* conidiation and spore dispersal while compromising maize growth and stress-related defense. Thus, high temperatures and drought stress are typically associated with AFL contamination (Cotty and Jaime-Garcia, 2007; Payne et al., 1986; Payne et al., 1988; Widstrom et al., 1990). To accurately assess mycotoxin risk and deploy pathogen-specific mitigation strategies, a clear understanding is needed of the association between mycotoxin outbreaks and environmental conditions, and fungal infection. High concentrations of mycotoxins can be present in maize grain even when obvious signs and symptoms of ear rot are absent. Therefore, determining contamination probability earlier in the season, prior to key developmental stages when maize is more susceptible to fungal infection (e.g., at flowering), would be useful for stakeholders by allowing them to implement different integrated pest management (IPM) strategies to avoid crop losses.

Previously published predictive models based on mechanistic and artificial intelligence (AI) algorithms were developed to forecast AFL and fumonisin (FUM) outbreaks in maize grown in Serbia and Italy (Leggieri et al., 2021; Liu et al., 2021). However, these models were neither trained with U.S. historic weather or mycotoxin data, nor have they been validated for prediction of maize AFL outbreaks in the U.S. One notable exception is the web-based Fusarium head blight (FHB) risk assessment tool that has been proven to be a valuable resource for wheat and barley growers in more than 30 U.S. states (U.S. Wheat and Barley Scab Initiative—https://www.wheatscab.psu.edu/). In a previous study, a mycotoxin predictive model based on U.S. farmer insurance claims due to AFL contamination was developed to forecast the possibility of future contamination as affected by environment (Yu et al., 2022). However, this study noted that prediction of AFL contamination based on selected insurance claims does not account for the variability of actual contamination levels throughout maize fields in the U.S. Another U.S. model, based on variable environmental scenarios in the state of Georgia, indicated the likelihood of AFL contamination events will increase in the future and mitigation strategies should be implemented immediately (Kerry et al., 2021).

Soil properties and land use potential have not been considered in previously developed predictive models for mycotoxin outbreaks in Georgia (Kerry et al., 2021) and other U.S. regions (Yu et al., 2022; Kerry et al., 2021; Abdelfatah et al., 2019), although it is known that these properties can impact maize growth and susceptibility to mycotoxin contamination (Castano-Duque et al., 2023; Castano-Duque et al., 2022; Branstad-Spates et al., 2024; Branstad-Spates et al., 2023). Recent predictive models, including our previous publications for Illinois (IL) (Castano-Duque et al., 2023) and Iowa (IA) (Branstad-Spates et al., 2024; Branstad-Spates et al., 2023) incorporated soil properties via a geospatially dynamic approach to quantify the contribution of these factors to historic mycotoxin outbreaks. These models demonstrated that in addition to pre- and post-planting weather factors, soil properties are significantly correlated with AFL and FUM contamination at harvest (Castano-Duque et al., 2023).

Due to the weather differences between the northern and southern maize-growing regions of the U.S., we developed AFL-focused models specifically for Texas (TX), using existing models from Illinois (IL) and Iowa (IA) as templates [21, 23, 24]. The published models incorporated geospatially dynamic weather input and soil properties. For the feature engineering of the TX models, we created a maize phenology model capable of determining average planting dates in each county. Additionally, we developed a new aflatoxin risk index (ARI) based on Ratkowsky growth equations (Ratkowsky and Reddy, 2017).

## 2 Materials and methods

### 2.1 Mycotoxin data

We used 14 years of historical AFL contamination data that included the years 2003, 2008–2009, 2012–2021 and 2024 (Table 1). Historic mycotoxin survey data for 2012–2021 were collected from a publicly available database (http://mycotoxinbmps.tamu.edu/mapsupdate.aspx, accessed February 10, 2021) that included AFL contamination levels at the county level throughout TX based on analyses by the Office of the Texas State Chemist (OTSC). The remaining AFL historic data we acquired (e.g., years 2003, 2008, 2009 and 2024) were based on AFL measurements from field samples collected by USDA-ARS colleagues. AFL survey data by county were used as the average per county per year for a total of 672 data points (ground truth). AFL contamination data were numerically categorized using 20 ppb (20 ng/g) as a threshold; therefore, contamination was labeled as high (> 20 ppb) or low (< 20 ppb). Selection of this threshold was based on the U.S. Food and Drug Administration’s AFL action level for AFL concentrations in food and feed.^1^ The AFL data were linked to all input features and divided into three groups: validation year data (Single year – 2013), training-set (70%) and testing-set (30%). The validation year dataset had a 13% incidence of AFL concentrations greater than 20 ppb. The validation dataset (from year 2013) had a total of 54 AFL ground truth data points, and the training datasets had 618 data points (after removal of 2013). The training and testing AFL datasets were skewed toward zero values, meaning high AFL events were considered outbreaks with low incidence (Castano-Duque et al., 2022). To generate a more balanced dataset prior to model training, we implemented the synthetic minority oversampling technique (SMOTE) (Torgo, 2011), using the *SMOTE* package in R, during data pre-processing. After performing the SMOTE balancing this data set had 773 AFL ground truth data points with an incidence of 310 high and 463 low contamination events, and 304 input features for weather (pressure [1–52 weeks], precipitation [1–52 weeks], temperature [1–52 weeks], relative humidity [1–52 weeks], soil moisture [1–52 weeks], and soil features) and 200 input features for ARI models (pressure [1–52 weeks], ARI [1–52 weeks], soil moisture [1–52 weeks], and soil features).

**Table 1:** Incidence of AFL contamination in TX maize in 2003, and from 2008 to 2021 and 2024.

Year	Variable	Aflatoxin (Modular)		Aflatoxin (ppb)
		High	Low	
2003	N-Counties	1.0	0.0	1.0
	Incidence/Mean	100%	0%	70.0
2008	N-Counties	7	6	13.0
	Incidence/Mean	54%	46%	53.0
2009	N-Counties	3.0	2.0	5.0
	Incidence/Mean	60%	40%	76.0
2012	N-Counties	22	39	61.0
	Incidence/Mean	36%	64%	27.0
2013	N-Counties	7	47	54.0
	Mean	13%	87%	9.6
2014	N-Counties	20	44	64.0
	Incidence/Mean	31%	69%	17
2015	N-Counties	9	53	62.0
	Incidence/Mean	15%	85%	10
2016	N-Counties	9	64	73.0
	Incidence/Mean	12%	88%	12
2017	N-Counties	16	52	68.0
	Incidence/Mean	24%	76%	20
2018	N-Counties	28	32	60.0
	Incidence/Mean	47%	53%	40
2019	N-Counties	15	62	77.0
	Incidence/Mean	19%	81%	14
2020	N-Counties	18	44	62.0
	Incidence/Mean	29%	71%	29
2021	N-Counties	6	57	63.0
	Incidence/Mean	10%	90%	8.3
2024	N-Counties	1	8	9.0
	Incidence/Mean	11%	89%	12

### 2.2 Output variables and correlation analysis

After binary categorization of output variables (high and low), we performed a pair-wise correlation analysis with a cut-off confidence intervals level of 0.95 in R (R Core Team, 2014). This correlation analysis was performed with all input features that were used in each of the three models that included weather data (average weekly precipitation, temperature, barometric pressure and humidity; soil properties; GPS centroids per county), AFLA-MAIZE-only (average weekly ARI from AFLA-MAIZE calculation methods and barometric pressure; soil properties; GPS centroids per county) and Ratkowsky-only (average weekly ARI from Ratkowsky calculation methods and barometric pressure; soil properties; GPS centroids per county).

### 2.3 Weather and soil features data collection

We aggregated daily meteorological weather and soil moisture (kg/m^2^) data to the county level in TX using the phase 2 North American Land Data Assimilation System (NLDAS-2) variable infiltration capacity model dataset (VIC) (Xia et al., 2012). NLDAS-2 data were obtained from NASA GES DISC (NLDAS, 2012; Gorelick et al., 2017) (accessed 8/5/2024). Texas counties were derived from the U.S. Census Bureau’s Topologically Integrated Geographic Encoding and Referencing system (TIGER) geospatial data (TIGER/Line® Shapefiles, 2022). Meteorological data at a spatial resolution of 0.125 degrees were obtained from NLDAS-2 and included total daily precipitation, mean daily temperature, minimum daily temperature, maximum daily temperature, mean daily specific humidity, mean daily barometric pressure. Mean daily soil moisture estimations were calculated from the raw hourly NLDAS-2 model derived product using the R terra package (Hijmans, 2025). Soil moisture data was collected from Layer 1 in the NLDAS-2 VIC model, in this data set the soil properties and soil layer depth vary with land cover-type in the model domain (Liang et al., 1994), meaning that soil moisture values for the upper Layer 1 do not represent a fixed depth interval, e.g., 0–30 cm, across Texas. Therefore, soil moisture values in Layer 1 represent the modeled upper most soil moisture layer that has varying thickness based on land cover-type, which are adjusted with model calibration in simulating runoff and baseflow components (Liang et al., 1994). A mean relative humidity value was calculated from mean daily temperature, mean daily specific humidity using mean daily barometric pressure implemented in the huss2hurs function from the R loadeR package (Iturbide et al., 2019; Bolton, 1980). Data were obtained for the period of 1 January 2003 to 31 December 2021, coinciding with the time frame of mycotoxin data collected from the TX counties selected for this study.

Data of soil features from arable land in TX were summarized by application of a filtering data mask layer to find mean values of soil properties in cultivated areas of each county. These features were used as inputs for the AI models to predict AFL outbreaks. The physical soil property features used in the mycotoxin model were water-holding capacity, saturated hydraulic conductivity, bulk density, and soil texture (i.e., sand, silt and clay content). Soil chemical property features were calcium carbonate content, cation exchange capacity, electrical conductivity, pH, and organic matter content. Estimates of the soil properties were determined at 800 × 800 m pixel resolution for various soil depth increments using measured values and interpolation techniques (Walkinshaw et al., 2022; O'Geen et al., 2017; Soil Survey Staff, Natural Resources Conservation Service, United States Department of Agriculture, 2012). The data mask layer was generated with a dataset acquired from the National Agricultural Statistics Service Cropland Data Layer (NASS CDL—https://nassgeodata.gmu.edu/CropScape/) (Boryan et al., 2011; Lark et al., 2017). Pixels, representing land within the NASS CDL that had been cultivated for more than 5 years (between years 2013 and 2022), were classed as arable (value 1), while pixels representing land that had been cultivated for less than 5 years were classed as non-arable (value 0), such as pastures, forests, urban spaces, brushlands, or other non-arable lands. Soil properties were then queried for all pixels with a value of 1 in the soil properties data layer (Walkinshaw et al., 2022) and summarized statistically by land area for each county.

The normalized difference vegetation index (NDVI) is a widely-used value, obtained from remote sensing reflectance data, for quantifying vegetation density and health (Rouse et al., 1973). To summarize time-series crop health and density for each TX county, a cropland data-layer mask was first generated for each year (from 2004 to present) to conduct target analysis of NDVI for cropped land only. For years 2004–2012, the NASS CDL was queried at its native pixel resolution of 30 × 30 m to determine cropping status by year, wherein pixels with any of the 106 crops reported within NASS CDL were coded as 1, and pixels with no crop were coded as 0, to generate a mask raster. For years 2013 through 2023, the NASS CDL provided an estimate of cultivated land for each 30 × 30 m pixel, making coding by crop type unnecessary. A mask was produced of value 0 for the class “non-cultivated” and value 1 for “cultivated” from the NASS CDL to guide the query of NDVI per county for these years. NASA’s Moderate Resolution Imaging Spectroradiometer (MODIS) program provides satellite-derived land-surface reflectance data from which NDVI can be calculated (Schaaf and Wang, 2015). To obtain NDVI values for each cropland pixel at a daily timestep, the MODIS Terra Daily NDVI dataset was queried within the Google Earth Engine for each cropped pixel of each county, and daily summaries of mean NDVI on cropland were tabulated for each county from 2004 to 2023 after masking by yearly cultivated land (Gorelick et al., 2017; Schaaf and Wang, 2015).

### 2.4 Phenology model for maize planting times

#### 2.4.1 Training data

Over the period of 21 years (2000–2020) (Marek et al., 2020), various crops (cotton, soybean, sorghum, corn, and sunflower) have been grown at USDA-ARS in Bushland, TX under a controlled, irrigated agricultural management practices, and the planting and harvest dates have been recorded. To generate a corn phenology model that estimates average planting dates, daily NDVI was extracted from the MODIS MCD43A4.006 dataset from the GPS coordinate location of the Bushland farm, using a single 463.313 meters pixel at the site. This dataset was used to build the planting date predictive model.

#### 2.4.2 Test data

To test the precision and statistical significance of the model, we used USDA-ARS data in Texas A&M Corn Variety Trials (Texas A&M AgriLife Research). Texas A&M Corn Variety Trials consist of 8–12 different sites throughout TX from 2018 to 2023, and the planting and harvest dates are documented. Daily NDVI was extracted from the MODIS MCD43A4.006 dataset from the GPS coordinate locations of each site during the maize growth from planting to harvest. This NDVI consists of a single 463.313 meters pixel at each site’s location. The variety trials were conducted independently at different locations and years, so they could be used objectively to measure modeling efficacy in predicting unseen data. These data specifically represent maize growth, as all the trial sites were exclusively planted with this crop.

#### 2.4.3 Outlier removal

To address outliers caused by fluctuations in satellite imaging, we implemented an algorithm to remove sudden changes in the data. In this algorithm, for $x_{i}$ ∈ X, where X = Time (unit: day) and $y_{i}$ ∈ Y, where Y = daily NDVI and i spans from January 1, 2000 to December 31, 2020 (with strict time progression), the point ( $x_{i},y_{i}$ ) is removed if: $\left|y_{i-1}-y_{i}\right|$ > 0.05.

#### 2.4.4 Model construction

For phenology model computations, MATLAB version R2023a was used (The MathWorks, Inc., 2023). Outliers in the NDVI data from the Bushland dataset were removed for each year. The data were then filtered to capture NDVI during the growth period for each year by retaining values from 30 days after the planting date until the harvest date. Subsequently, a 3rd-degree polynomial was fitted to the data for each year using the least squares method. As previous work in the literature (Kalácska et al., 2005; Irmak et al., 2008) has illustrated, the growth curve of crops is well represented by using a 3rd-degree polynomial. The leading coefficient is kept positive to ensure a root relatively near the planting date.

From these polynomials, the local maximum of the polynomial and the minimum x-value, such that y = 0, was extracted for each year and location. As shown in previous studies, the day of theoretical zero NDVI correlates with the sprouting date of crops (Gao et al., 2021), and the day of maximum NDVI value can be used as an indicator for determining the planting date (Deines et al., 2023). In our study, we found the day of theoretical zero NDVI, paired with the rate of NDVI growth (rate in respect to functions zero to functions local maximum), as useful indicators for determining the planting date.

Multiple linear regression was used to optimize these two variables for prediction of planting date, where $X_{1}$ is the minimum x-value extracted from the values when y = 0, and $X_{2}$ is the local maximum NDVI of the cubic function divided by the number of days from $X_{1}$ . Equation 1 presents the result of the regression algorithm:

(1) $$\mathrm{Planting}\;\mathrm{Date}=0.69X_{1}-1050.3X_{2}+8.37$$

#### 2.4.5 Performance evaluations

To evaluate the model’s performance, we used mean absolute error (MAE), mean absolute error standard deviation (*R*^2^) and root mean square error (RMSE).

#### 2.4.6 County planting date predictions

Daily average NDVI for each county was collected for each pixel identified as land used for cultivation, representing all types of crops grown each year across TX, for each year from 2008 to 2022. The average NDVI for the pixels in each county’s outliers were removed based on the time series continuity. The time period for crop growth for each year’s data was established by analyzing sequential data points surrounding the day of maximum NDVI. Days before and after this peak were included until reaching an NDVI value below the annual mean for that year and county. The remaining NDVI for each year and county was then fit to a 3rd-degree polynomial. After computing the 3rd-degree polynomial for each county and year, the planting date was determined using Equation 1. The maximum data point for counties was identified within the period of February 1st to August 1st; this ensured we would capture the earliest growing period in instances where certain counties plant crops twice a year.

### 2.5 Growth and AFL experiments using the Ratkowsky model

#### 2.5.1 Growth chamber experiments using variable temperature

One *A. flavus* strain, NRRL 3357 (Nierman et al., 2015), herein called AF3357 (1 × 10^4^ CFU/mL) was single point inoculated onto the center of potato dextrose agar (PDA, DIFCO) plates and incubated in darkness for 7d at 5, 10, 15, 20, 25, 30, 35, 40 and 45°C (5 replicate plates per temperature). Fungal growth was measured daily as colony diameter, and on day 7 the cultures were prepared for assessment of AFL production.

#### 2.5.2 Aflatoxin measurements via UPLC analysis

From each AF3357 colony, five agar plugs (6 mm) were excised and placed in 200 mL glass vials for metabolite extraction with acetonitrile:water:formic acid (80:19:1, v/v/v, 1 mL). The contents were incubated on an orbital shaker (200 rpm) for 2 h at room temperature. The extracts were then centrifuged to pellet particulates, and the particulate-free extracts were transferred to clean tubes and analyzed (1 μL injections) using a Waters ACQUITY UPLC system (40% methanol in water, BEH C18 1.7 μm, 2.1 mm × 50 mm column) with fluorescence detection (Ex = 365 nm, Em = 440 nm). Some samples needed dilution to avoid saturating the detector. Identification and quantification utilized an analytical standard of AFL B_1_ (AFB_1_) purchased from Sigma-Aldrich (St. Louis, MO, United States). AFB_1_ content was expressed in parts per billion or PPB (ng/g agar).

#### 2.5.3 Data analysis of fungal growth and AFL production

Fungal growth was calculated by fitting a Baranyi growth model (Baranyi and Roberts, 1995) to the data for each temperature regime using the *growthrates* package (Hall et al., 2014) in R (R Core Team, 2014). The Baranyi model considers that there is a lag phase for growth and is based on two differential equations (Baranyi and Roberts, 1995). Using this model fitting, we were able to determine the growth parameters of initial growth (y_0_), growth rate (𝜇_max_) and maximum growth (K) for each temperature tested. We used the growth rate values that had *R*^2^ > 0.95 to fit a second growth model to determine growth rate as a function of temperature by using the Ratkowsky equation (Ratkowsky and Reddy, 2017) in R (Equation 2):

(2) $$\mathrm{rate}={\left\{a*\left(t-\mathrm{tmin}\right)\right\}}^{2}*{\left(1-e^{\left(b*\left(t-\mathrm{tmax}\right)\right)}\right)}^{2}$$

For fungal growth rate, the constants were: a = 9.117, b = 0.0001842, t_min_ = 5.41 and t_max_ = 47.4. For AFL production rate, the constants were: a = 53.36, b = 0.0003373, t_min_ = 11.62 and t_max_ = 38.04.

### 2.6 Aflatoxin risk index

Two different ARIs were generated using equations to calculate fungal growth and AFL production from the AFLA-MAIZE method (Castano-Duque et al., 2023) or the Ratkowsky method (Equation 3). An AFL risk index was calculated using the AFLA-MAIZE model generated by Battilani et al. (2013), and this method used the beta equations for modeling fungal growth and AFL production. To assess the efficacy of the AFLA-MAIZE derived mechanistic model, we compared it to a Ratkwosky derived mechanistic model. The Ratkwosky derived model is based on a temperature-dependent equation that considers the asymmetrical, instantaneous growth of *A. flavus* (Ratkowsky and Reddy, 2017; Ratkowsky et al., 1983; Shi et al., 2017). The Ratkwosky growth equation has been described as one of the best tools for modeling temperature dependence of fungal growth (Zwietering et al., 1991; Dey et al., 2020).

(3) $$\begin{array}{l} ARI=\mathrm{growth}\;\mathrm{or}\;\mathrm{weighted}\_\mathrm{growth}\;x\;AFL\;\mathrm{or}\;\\ \mathrm{weighted}\_AFL\;x\;\mathrm{dispersal}\;x\;\\ \left(1+ECB\_\mathrm{damage}\right) \end{array}$$

For daily ARI calculations using the AFLA-MAIZE method, fungal growth was estimated as described by Castano-Duque et al. (2023). For calculating the ARI from the Ratkowsky method, we used Equations 2, 3. Weighted fungal growth (10% of original growth) was generated using the cut-off values calculated from the phenology model for planting time and 120 days for harvest time (Texas Corn Producers, 2024). These dates were included by calculating the weighted fungal growth and AFL production for both AFLA-MAIZE and Ratkwosky ARIs (Equation 3). The weighted growth is an assumption in our models, it considers that fungal growth, prior to maize planting and after harvest, was 90% lower based on availability of maize substrate for the fungi to live. The daily AFL production index was calculated as described by Castano-Duque et al. (2023) for the AFLA-MAIZE-ARI or using Equation 2 of the Ratkowsky-ARI. For both ARIs, spore dispersal was set as an ON/OFF switch (Castano-Duque et al., 2022; Van der Fels-Klerx et al., 2019) under specific precipitation and relative humidity conditions. We assumed no spore dispersal (OFF) if there was any precipitation and/or if the relative humidity was greater than 80%; otherwise, there was dispersal (ON). This inference was based on a previous study that identified a negative prognosis of spore dispersal under positive rain conditions (Ji et al., 2023). In our model, we did not consider wind speed although we understand the importance it has for dispersal of *Aspergillus* (Segers et al., 2023). Nevertheless, only precipitation and relative humidity were used in this model due to the consistency of daily historic records throughout the geographical regions included in this case study. Insect damage was calculated for European corn borer damage by using growing degree days of the insect (T_base_ = 6°C and T_cut_ = 30°C) and the logistic equation (Maiorano et al., 2009) as described by Castano-Duque et al. (2023).

For any missing values in the weekly ARI, we used multivariate imputation with chained equations, specifically the Predictive Mean Matching (pmm) method in R (R Core Team, 2014; van Buuren and Groothuis-Oudshoorn, 2011). Finally, we linked the AFL data to the feature dataset to create data points and 153 features or predictors. We lagged the weekly inputs starting 6 months prior to the first year of AFL data available (2003). Thus, single year validation was performed using weather data that included the last 6 months of 2012 and the first 6 months of 2013 meaning that a yearly prediction can be performed on week 26 (End of June, beginning of July). There was a total of 597 AFL data points, with 148 input features, for the AFLA-MAIZE and Ratkowsky ARI based models, and 252 input features for weather models.

### 2.7 Local spatial autocorrelation assessment using Getis-Ord Local Gi Test

Local spatial autocorrelation was also assessed using the Getis-Ord Local Gi Test (Gi*) among county aggregated values of meteorological variables, soil moisture levels, soil properties and ARIs in determining if values, relatively high or low, were spatially clustered across the counties. Spatial autocorrelation is a measure of how values for a parameter, e.g., temperature, are related in space (Burt et al., 2009). Values of Gi* were assessed through a workflow described by Leary (2023) using the *sfdep* and *spdep* R packages. The Gi* statistic specifically tests whether relatively high or low values, from a range of values, are clustered in space. Additionally, Gi* also tests whether clustering of high, moderate and low values is considered statistically significant at *α* thresholds of 0.1, 0.05 and 0.01, respectively, against the null hypothesis that values are randomly distributed in space (Getis and Ord, 1992; Ord and Getis, 1995). We note as part of the implementation described by Leary (2023), the TX counties not used in this study were removed prior to calculating Gi*.

### 2.8 Gradient boosting model

A GBM (standard and adaboost) was used to predict mycotoxin contamination since it allows for determining the importance of input features on the output variable. The GBM software package in R that we used included Freund and Schapire’s AdaBoost algorithm and Friedman’s gradient boosting machine (Friedman, 2001). For performing GBM, we removed county and year from the dataset so that only data from 2013 was used for validation. Next, we balanced the data, using the synthetic minority oversampling technique (SMOTE) from the DMwr package in R (R Core Team, 2014), to create a new, balanced dataset with oversampled observations from the high contamination level class. The balanced data were partitioned using a 70:30 ratio. We performed GBM with standard stochastic and adaboost distribution methods, followed by hyperparameters fine-tuned using a grid search method applied on the model’s interaction depth, shrinkage, and minobsinnode. We performed ten cross validation folds. Finally, we computed the importance values (i.e., variable relative influence) for each predictor in the model by reducing the sum of squared error due to the splits on that predictor, then averaging the improvement made by each variable across all the trees, to determine the relative effect (Friedman, 2001). We generated a confusion matrix, computing overall and specific statistics by class.

### 2.9 Neural network model

A NN was selected (nnet) as a secondary modeling method because of its high performance in the prediction of rare events (Zamani and Kremer, 2013; Gibson and Kroese, 2022) by using caret software package in R. For training the NN, we again removed prior year county data so that only 2013 data was used for validation. Data without the validation set was balanced using the SMOTE method. Balanced data were partitioned at the 70:30 ratio for training and testing. The mean and standard deviation scaling methods were applied to each input feature of the training, testing and validation datasets. For training purposes, the best NN architecture parameters (i.e., hidden layers and neurons) were determined using a grid search method. The model’s performance was assessed using a test dataset (30% of the total data) and single year (2013) validation datasets for evaluating the accuracy, sensitivity and specificity.

### 2.10 Single-year validation

To perform prediction analysis using GBM and NN, we used a single year, 2013, because this year had an incidence of 13% (Table 1) for high AFL contamination events (7 out of 54 observations). Validation involved the best fit of GBM-standard, GBM-adaboost, and nnet for weather, AFLA-MAIZE-ARI and Ratkowsky-ARI models. All the input features for validation years were prepared as previously described in the methods section for the training data.

## 3 Results

### 3.1 Phenology model for planting times

**Figure 1:**
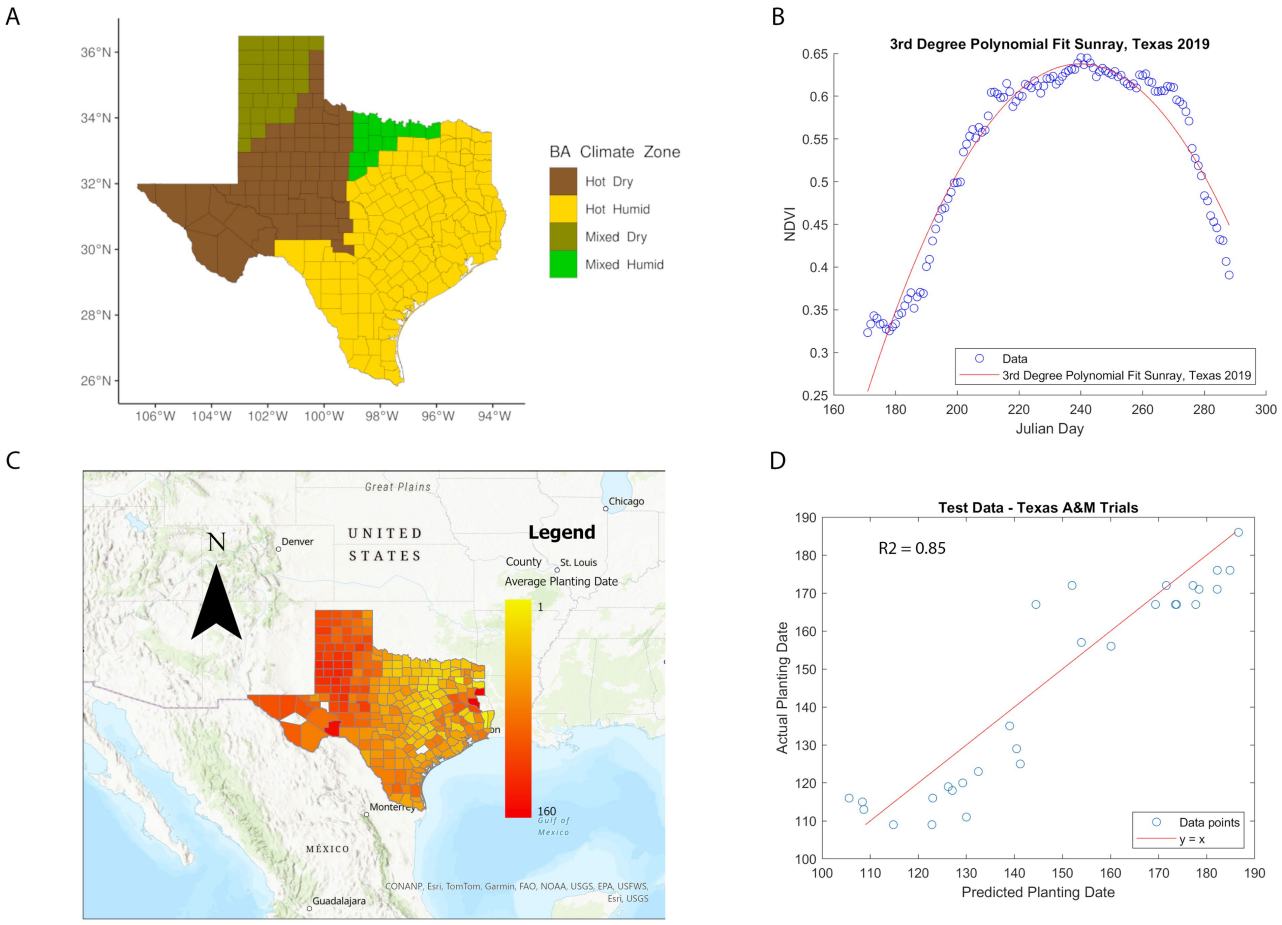
Texas climate zones and phenology model. **(A)** BA climate zone geospatial distribution in TX counties. The Y-axis represents latitude, X-axis longitude. Brown: Hot-Dry, yellow: Hot-Humid, olive: Mixed-Dry, light-green: Mixed-Humid. **(B)** Third degree polynomial fit of NDVI data. The blue points represent the daily average NDVI for cultivated land in Sunray, Texas, in 2019. The red line is a third degree polynomial fit to these points. **(C)** Planting dates from phenology model. Each Texas county is color-coded based on the average planting date for cultivated land from 2008 to 2022. White counties indicate insufficient data for an estimate. Yellow counties represent early planting dates, while red counties correspond to later planting dates, up to Julian day 160. **(D)** Performance of phenology model on testing data. The blue points represent the predicted planting dates versus the actual planting dates for 29 testing data points used to validate the planting date prediction model. The red line represents the y = x line, where alignment of points would indicate a perfect model. The R^2^ value of the model is 0.85.

Implementation of ARI calculations used the average planting dates, from 2008 to 2022, for different counties in TX, providing a maximum of 15 years. Not all counties reached this span of time and there were instances where no pixel values represented cultivated land in a county (for certain years). Also, in a few cases, the model predicted a planting date being in the year prior, which we excluded from the average calculation. Out of the counties calculated, 81% of averages included at least eight or more years. Planting dates for cultivated lands in TX counties were calculated using Equation 1. Our results show that average planting dates in TX ranged from January to June. The average planting date per county is shown in Figure 1, in which counties without color represent uncultivated land, or in the years where pixels were present, the predicted planting date fell in the previous year, which has been excluded from the averages. The model’s mean prediction error for planting dates was 6.8 days for the training data from Bushland, TX and 8.6 days for the new data from the A&M Variety Trials. The R-squared value for our test data set was 0.85. These metrics showed the model was a good predictor for new data from various regions in TX. The phenology model showed that the hot-dry and a large portion of the hot-humid region has planting dates between February and March, and harvest between June and July. Notably, there is a transect of early planting dates in North Central TX, corresponding to the Blackland Prairie region—an area known for its extremely fertile soil, rich in organic matter, and ideal for farming.

### 3.2 AFLA-MAIZE and Ratkowsky ARIs

Mycotoxin contamination data had notable incidences of high AFL levels (>20 ppb), from 10 to 100% (Table 1), with variable number of annual AFL data points reflecting the total mycotoxin tests per county (N_min_ = 1 to N_max_ = 77). We determined, by using the Ratkowsky equation, that the modeled growth rate of *A. flavus* had limits, with minimum and maximum temperatures of 5.41°C and 47.4°C, respectively, and the optimal temperature for maximum growth rate was between 20°C and 25°C (Supplementary Figure S1). Using the Ratkowsky equation, we also determined that AFL production had limits, with respective minimum and maximum temperatures of 11.6°C and 38°C, and the optimal temperature for AFL production was 25°C (Supplementary Figure S1). The Ratkowsky’s minimum and maximum limit values differed ±1°C. Compared to AFLA-MAIZE’s values of fungal growth and AFL production, AFLA-MAIZE’s fungal growth temperature ranges showed a minimum of 5°C and maximum of 48°C, while AFL production temperature ranges showed a minimum of 10°C and maximum of 47°C. It is key to note that the fungal strain used to generate the AFLA-MAIZE values was not AF3357, which was used to generate the Ratkowsky values.

Comparing the daily ARI weighted values by week and by climate zone generated by AFLA-MAIZE and Ratkowsky equations (Supplementary Figure S2), we noticed that the range of quantiles and median of daily values by week was more variable in the Ratkowsky model compared to the AFLA-MAIZE model, and the overall distribution of daily ARI by climate zone was similar between the two mechanistic models (Supplementary Figure S2). The differences between ARIs from AFLA-MAIZE and Ratkowsky models can be observed in the weekly representation of Anderson County in 2008 and 2013 (Supplementary Figure S2). For the Ratkowsky model, end-of-year time points showed higher values than AFLA-MAIZE and most likely linked to the ±1°C variations in minimum and maximum ranges of fungal growth and AFL production limits.

### 3.3 GBM and NN

#### 3.3.1 Weather-centric models

**Figure 2:**
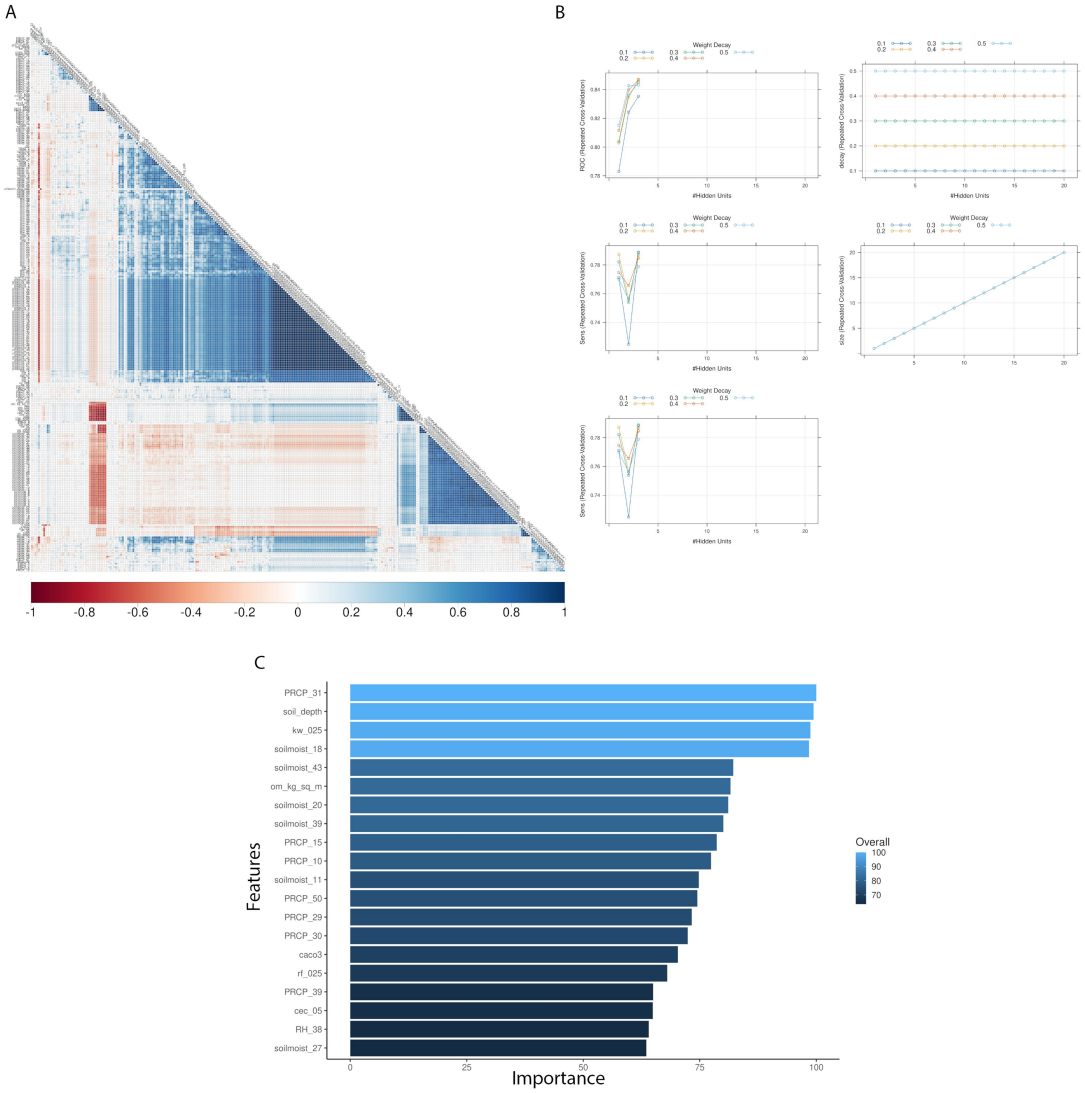
Texas AFL nnet model using weather-only input features. **(A)** Pair-wise correlation analysis of input variables used in the nnet model; **(B)** Results of fine tuning parameters (size and decay) of the nnet model by using cross-validation; **(C)** Top 20 influential input features and overall influence over the nnet model in the prediction of AFL. The correlation is depicted from positive (blue) to negative (red), with blank squares representing non-significant *p*-values of correlation between variables. For the correlation analysis, the *p*-value cut-off was 0.05, and the confidence level was 0.95. The blue hue in bar-plots represents relative influence of the input variables, with light blue high and dark blue low influence levels.

**Figure 3:**
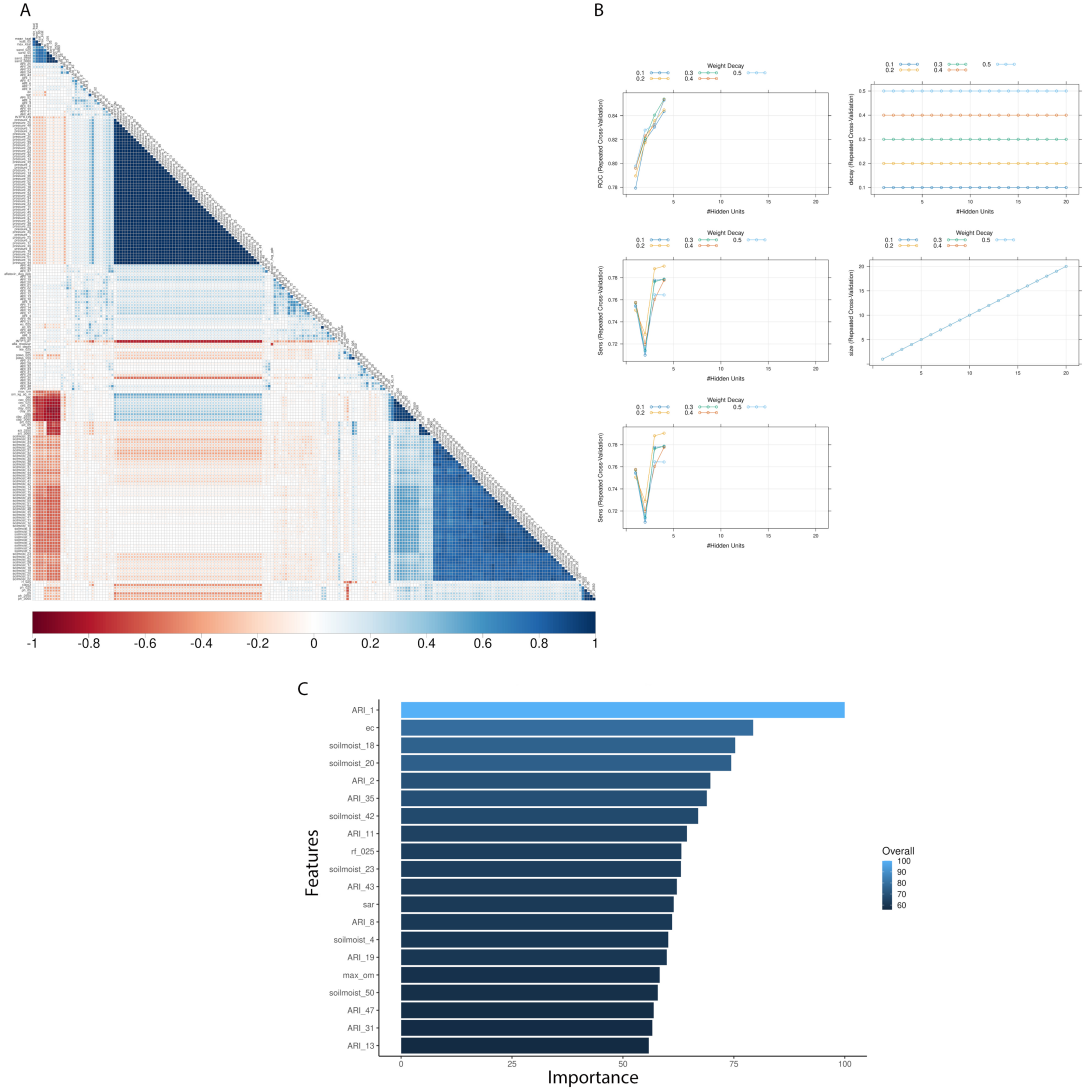
Texas AFL nnet model using AFLA-MAIZE ARI engineer input features. **(A)** Pair-wise correlation analysis of input variables used in the nnet model; **(B)** Results of fine tuning parameters (size and decay) of the nnet model by using cross-validation; **(C)** Top 20 influential input features and overall influence over the nnet model in the prediction of AFL. The correlation is depicted from positive (blue) to negative (red), with blank squares representing non-significant *p*-values of correlation between variables. For the correlation analysis, the *p*-value cut-off was 0.05, and the confidence level was 0.95. The blue hue in bar-plots represents relative influence of the input variables, with light blue high and dark blue low influence levels.

Pairwise correlation analysis of input features for the weather-centric models showed high positive correlation within weekly pressure, relative humidity and soil moisture variables showing that these variables are autocorrelated (Figure 2). To consider this high level of autocorrelation, we used both GBM and NN and performed grid fine-tuning of the parameters for all models. All models showed >90% balanced accuracy in the test data sets (Supplementary Tables S1, S2) and the nnet model showed the highest validation-set single year accuracy with 51% (Table 2; Supplementary Tables S1, S2). For single-year validation had a total of 54 data points, of which seven were labeled as high AFL levels (> 20 ppb), the nnet model correctly classified three of the seven high contamination events, and it incorrectly classified 18 out of 47 low contamination events (Table 3; Supplementary Table S3). The top influential input features of the nnet weather model were precipitation (weeks 10, 15, 30, 31, 39, and 50), relative humidity (week 38) and soil moisture (week 27) (Figure 2; Supplementary Figure S3; Supplementary Table S4). Additionally, soil properties included depth (cm), erodibility (kw between 0 and 25 cm), organic matter (kilograms/meter^2^), calcium carbonate (kilograms/meter^2^), as well as percentage of rock fragments and cation exchange capacity at depths between 0 and 25 cm (cmol/kg) (Figure 3; Supplementary Table S4).

**Table 2:** Summary statistics of best AFL models for test-set and single year-set generated by using weather specific inputs (nnet), AFLA-MAIZE ARI (nnet), and Ratkowsky ARI (nnet).

	Weather*	AFLA-MAIZE-ARI	Ratkowsky-ARI
Differences of inputs	Soil properties	Soil properties	Soil properties
	Barometric pressure	Barometric pressure	Barometric pressure
Model	nnet	nnet	nnet
Structure	Size = 3	Size = 4	Size = 4
	Decay = 0.2	Decay = 0.3	Decay = 0.3
	cv. = 10 with 7 repeats	cv. = 10 with 7 repeats	cv. = 10 with 7 repeats
Accuracy	0.57	0.74	0.74
95% CI	(0.4321, 0.7077)	(0.6035, 0.8504)	(0.6035, 0.8504)
No information rate	0.87	0.87	0.87
*p*-value [Acc > NIR]	1.00	1.00	1.00
Kappa	0.01	0.16	0.29
McNemar’s test *p*-value	<3e-3	0.18	0.02
Sensitivity	0.43	0.43	0.71
Specificity	0.60	0.79	0.74
Pos Pred value	0.14	0.23	0.29
Neg Pred value	0.88	0.90	0.95
Prevalence	0.13	0.13	0.13
Detection rate	0.06	0.06	0.09
Detection prevalence	0.41	0.24	0.31
Balanced accuracy	0.51	0.61	0.73

* Weather parameters were temperature, precipitation and relative humidity, these parameters were used to feature engineer ARI. Test-set used was 30% of the data and single year validation-set was 2013.

**Table 3:** Contingency tables and number of data points of best AFL models for single year-set and test-set by using weather specific inputs (nnet), AFLA-MAIZE-ARI (nnet) and Ratkowsky-ARI (nnet).

Model	Prediction	Reference	
		High	Low
Weather-nnet*	High	3	18
	Low	4	29
AFLA-MAIZE-nnet	High	3	10
	Low	4	37
Ratkowsky-nnet	High	5	12
	Low	2	35

		Test-set	Validation-set (Single year)
*N*	High	93	7
	Low	138	47
	Total	231	54
Percentage	High	40	13
	Low	60	87

* Weather parameters were temperature, precipitation and relative humidity, these parameters were used to feature engineer ARI. Test-set used was 30% of the data and single year validation-set was 2013.

#### 3.3.2 AFLA-MAIZE-ARI models

Pairwise correlation analysis of input features for the AFLA-MAIZE-ARI model showed high positive correlation among pressure and soil moisture variables, while showing a negative correlation between soil properties and soil moisture. The model with the highest accuracy using the AFLA-MAIZE ARI was nnet (test-set 93% and validation-set 61%), having an interaction size of 4 and decay of 0.3 (Figure 3; Table 2; Supplementary Table S1). For the single-year validation, the nnet model was able to correctly classify three of the seven high contamination events and it incorrectly classified 10 out of 47 low contamination events (Table 3; Supplementary Table S3). Among the top 20 input features, those with highest importance were the AFLA-MAIZE-ARI (weeks 1, 2, 8, 11, 13, 19, 31, 35, 43 and 47), soil moisture (weeks 1, 4, 20, 23, 42 and 50), soil properties (electrical conductivity in decisiemens/meter), percentage of rock fragments at depths between 0–25 cm, sodium adsorption ratio, and maximum organic matter (percent by weight) (Figure 3; Supplementary Table S4).

#### 3.3.3 Ratkowsky-ARI models

**Figure 4:**
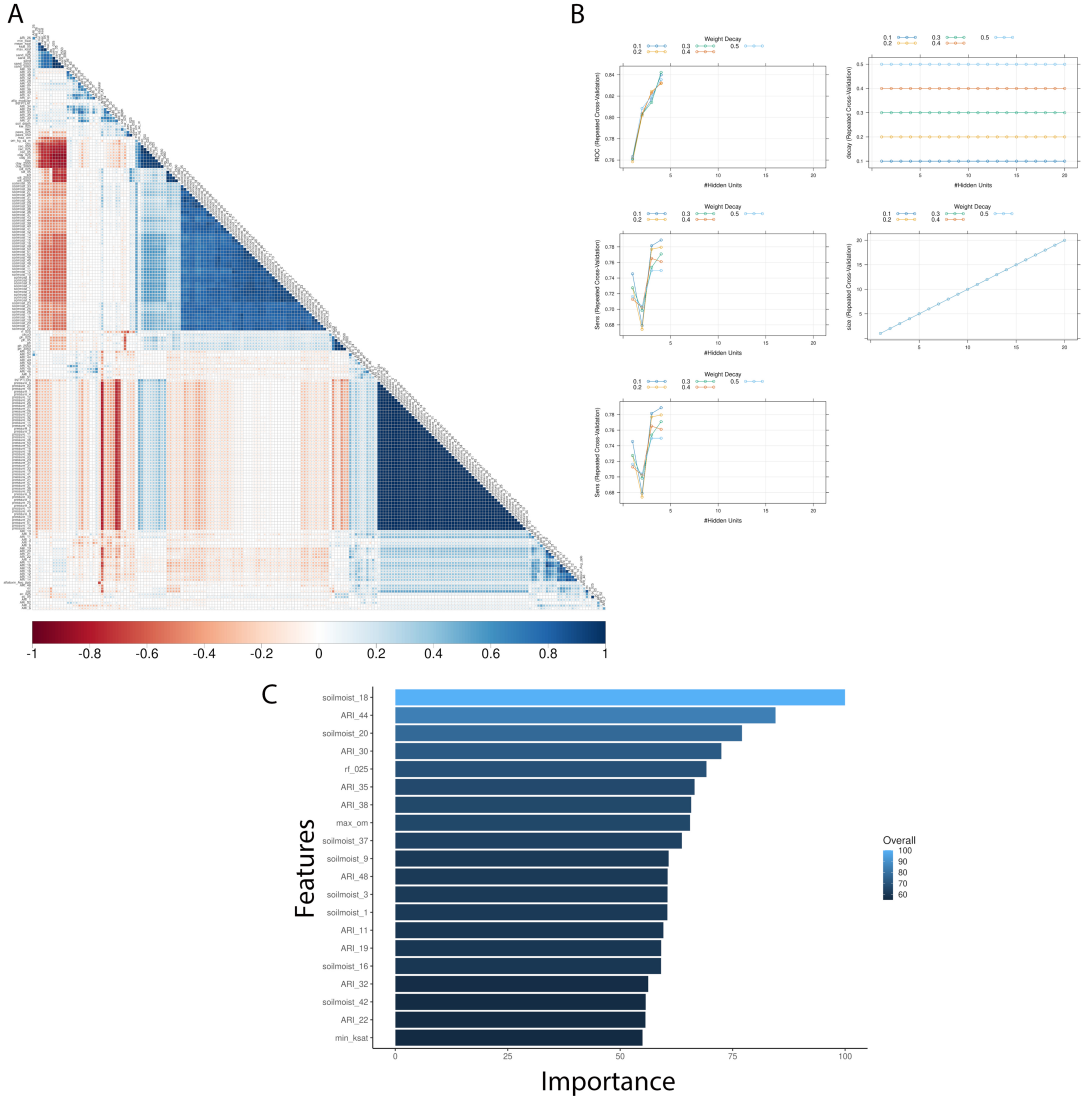
Texas AFL nnet model using Ratkowsky ARI engineer input features. **(A)** Pair-wise correlation analysis of input variables used in the nnet model; **(B)** Results of fine tuning parameters (size and decay) of the nnet model by using cross-validation; **(C)** Top 20 influential input features and overall influence over the nnet model in the prediction of AFL. The correlation is depicted from positive (blue) to negative (red), with blank squares representing non-significant *p*-values of correlation between variables. For the correlation analysis, the *p*-value cut-off was 0.05, and the confidence level was 0.95. The blue hue in bar-plots represents relative influence of the input variables, with light blue high and dark blue low influence levels.

**Figure 5:**
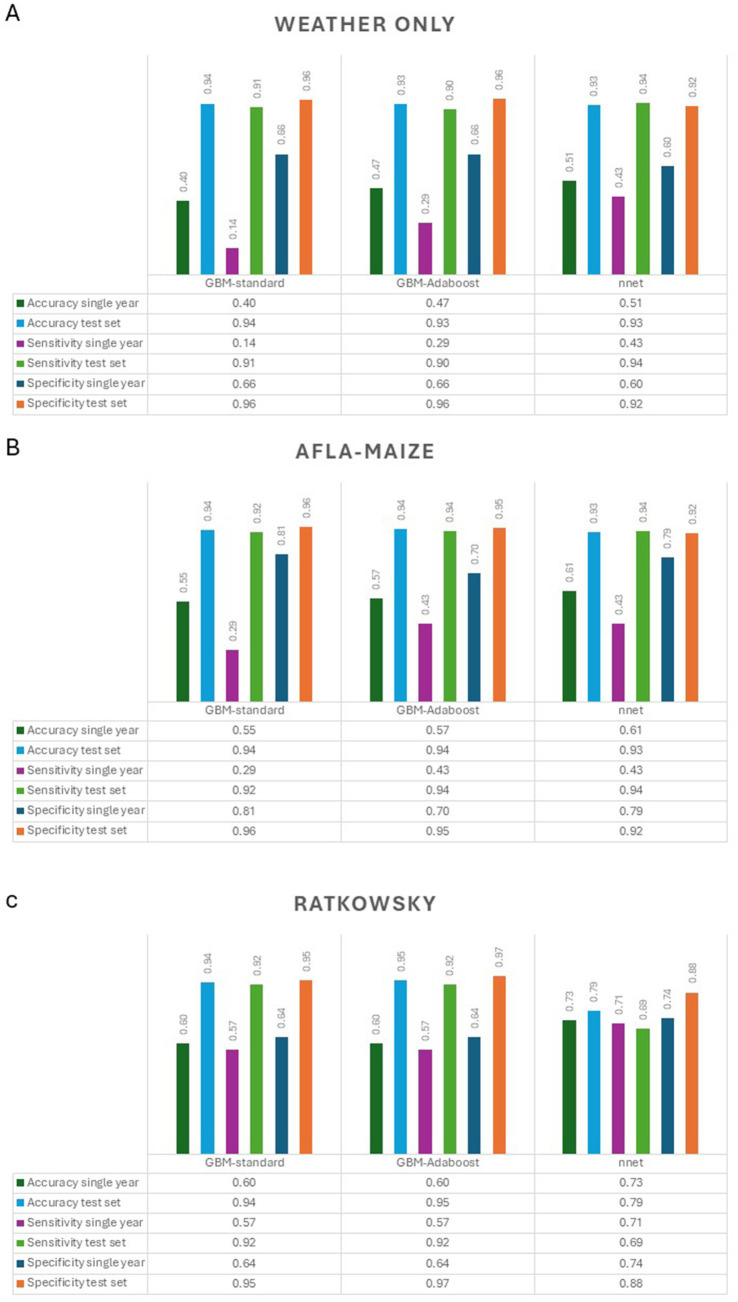
Summary of accuracy, sensitivity and specificity of the models (GBM-standard, GBM-adaboost, and nnet) used to predict AFL outbreaks in Texas. **(A)** Weather only input features, **(B)** AFLA-MAIZE ARI input, and **(C)** Ratkowsky ARI input.

Pairwise correlation analysis of input features for the Ratkowsky-ARI models showed high positive correlation among pressure and relative humidity variables, but a negative correlation among soil variables (Figure 4). From the three different models tested (GBM-standard, GBM-adaboost, and nnet), both GBM-standard and GBM-adabost showed >90% balanced accuracy in the test set and about 60% accuracy in the validation set (Supplementary Tables S1, S2). The nnet architecture had a size of 4 and decay of 0.3 (Figures 4, 5; Table 2; Supplementary Table S1) and showed the highest single-year validation values for accuracy (73%), sensitivity (71%) and specificity (74%) (Table 2; Supplementary Tables S1, S2). For the single-year validation, the nnet model was able to correctly classify five of the seven high contamination events and it incorrectly classified 12 out of 47 low contamination events (Table 3; Supplementary Table S3).

**Figure 6:**
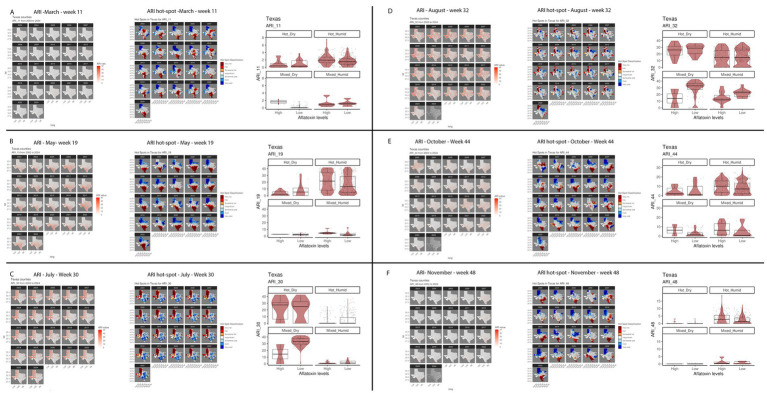
Geospatial distribution of top influential ARI from Ratkowsky-ARI nnet model and their relationship with AFL contamination levels in TX. **(A)** Week 11 (March); **(B)** week 19 (May); **(C)** week 30 (July); **(D)** week 32 (August); **(E)** week 44 (October); **(F)** week 48 (November). In each panel left – geospatial distribution of weekly average precipitation; middle – hotspot geospatial distribution of soil property; right – Soil property in relation with AFL levels by BA-climate zone. Maps of geospatial distribution of the weekly ARI are shaded in red from 2003 to 2023 or 2024 for each specific week, the y-axis is latitude, and the x-axis is longitude. Red and blue color palette of geospatial hot-spot analysis used the historic mean of gi-value for weekly ARI as the middle point scale, red hues are gi-values above the historic mean, and blue hues are below the historic mean. Hot-spot specific red/blue hues are classified by the level of significance of the p-folded value: “very hot/cold” < =0.01, “hot”/“cold” < = 0.05, “somewhat hot/cold” < = 0.1. Box–Whisker plot depicts the maximum (25th – 1.5 * interquartile range “IQR”) and minimum [75th percentile +1.5 *interquartile range (IQR)], and the Box–Whisker plot depicts median, first (25th percentile) and third (75th percentile) quantiles distribution, each panel represents an ecoregion of Texas (Hot-dry, hot-humid, mixed-dry, mixed-humid); For AFL classification, high is >20 ppb, and low ≤20 ppb. The violin plot is shaded in red and depicts the density distribution of the weekly average ARI and levels of mycotoxin contamination; and the gray dots depict each data point.

**Figure 7:**
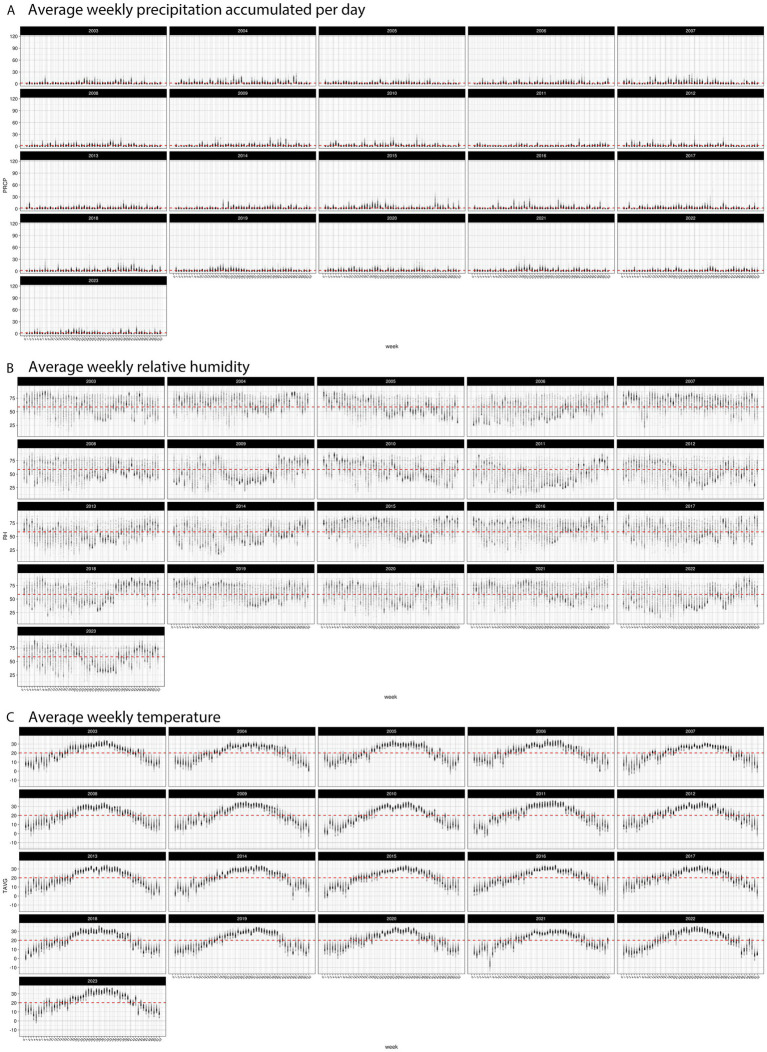
Distribution of weekly weather factors in TX from 2003 to 2024 **(A)** average precipitation (cm), **(B)** average relative humidity, and **(C)** average temperature. Red line indicates the historic average for each specific weather factor.

The top influencer features from the Ratkowsky-ARI nnet model include the ARIs in weeks 11, 19, 22, 30, 32, 35, 38, 44 and 48. We tested the geospatial relationship of specific regions in TX with the Ratkowsky-ARI and determined that in most of the significantly influencing weeks, high ARI correlates with high AFL contamination in the hot-dry, hot-humid, and mixed-humid regions (Figure 6). Unexpectedly, historical data for these weeks showed there was a very high hot-spot in the mixed-dry zone (TX panhandle) in week 30 and 32, and in these 2 weeks there was a negative relationship between AFL contamination levels and ARI only in mixed-dry zone. Interestingly, in these 2 weeks in the mixed-dry zone the average temperature followed a cold-spot pattern (low temperature averages) and the average precipitation was either non-significant or a hot-spot (high rain averages) (Figures 6, 7; Supplementary Figure S3). For some years, during weeks 30 and 32, a switch occurred from cold to hot-spots where the temperature was higher than the years where there was no switch (Supplementary Figure S4). The last weather and soil related feature in the Ratkowsky-ARI nnet model, among the top significant influencers, was soil moisture in weeks 18 (April) and 20 (May) (Supplementary Figure S4). We observed that throughout the history of climactic geospatial data, there was a recurrent hot-spot in limited areas among the hot-humid, hot-dry and mixed-humid zones (Supplementary Figure S4). We detected a negative correlation between high AFL levels and high soil moisture in the hot-dry and mixed-humid zones (for weeks 18 and 20) and a positive correlation in mixed-dry in week 18 (Supplementary Figure S4).

**Figure 8:**
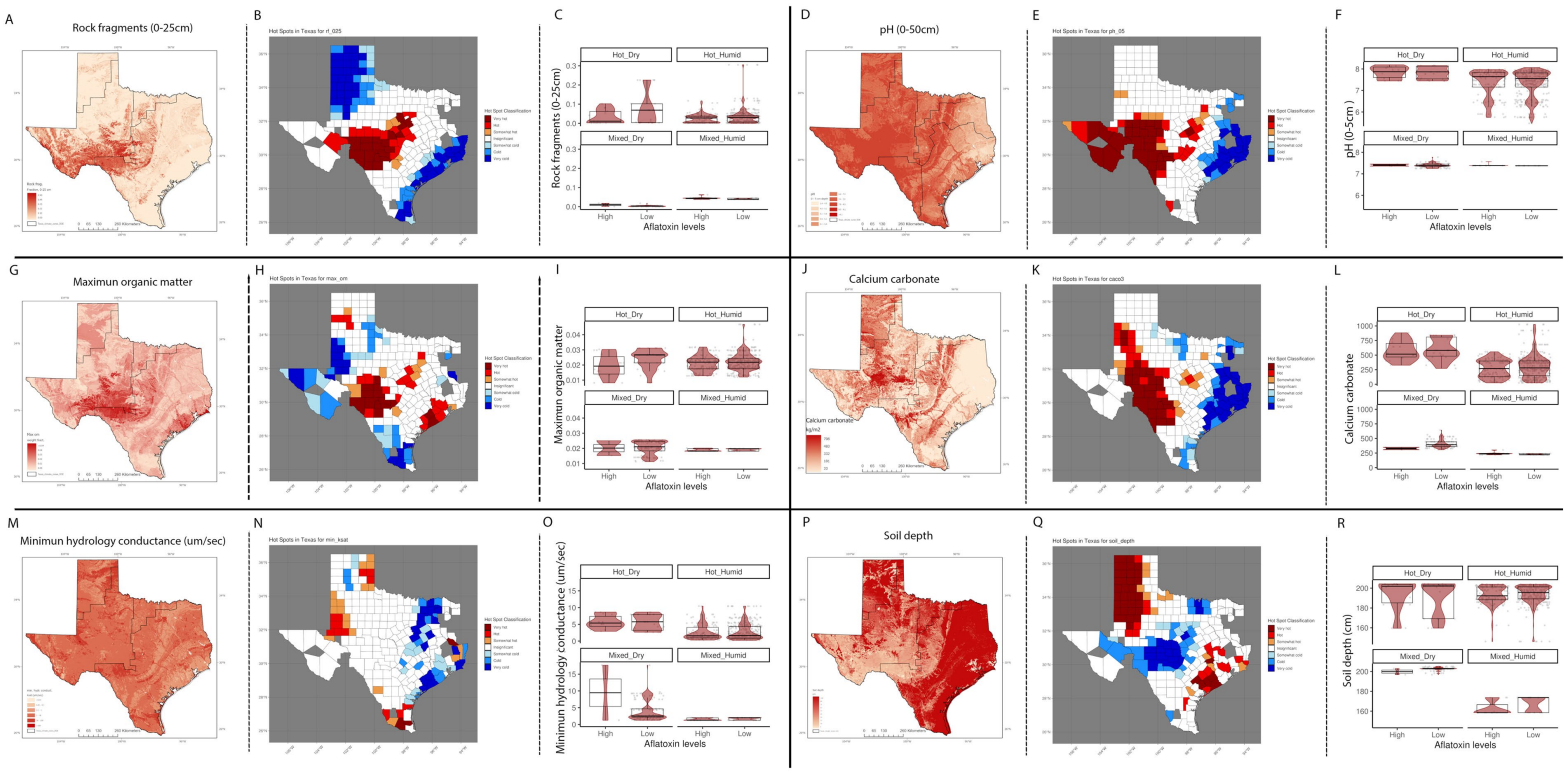
Geospatial distribution of top influential soil properties from Ratkowsky-ARI nnet model and their relationship with AFL contamination levels in TX. Rock fragments from 0 to 25 cm depth **(A)** distribution in TX, **(B)** hot-spots, **(C)** box-plots distribution by climate zone; pH from 0 to 50 cm depth (percentage by weight) **(D)** distribution in TX, **(E)** hot-spots, **(F)** box-plots distribution by climate zone; maximum organic matter (weight fraction) **(G)** distribution in TX, **(H)** hot-spots, **(I)** box-plots distribution by climate zone; calcium carbonate – CaCo_3_ **(J)** distribution in TX, **(K)** hot-spots, **(L)** box-plots distribution by climate zone; minimum hydrology conductance (μm/s), **(M)** distribution in TX, **(N)** hot-spots, **(O)** box-plots distribution by climate zone; soil depth (cm) **(P)** distribution in TX, **(Q)** hot-spots, **(R)** box-plots distribution by climate zone. Maps of geospatial distribution of each soil property are shaded in red, and the y-axis is latitude, and the x-axis is longitude. Red and blue color palette of geospatial hot-spot analysis used the mean of gi-value for each soil property as the middle point scale, red hues are gi-values above the mean, and blue hues are below the mean. Hot-spot specific red/blue hues are classified by the level of significance of the p-folded value: “very hot/cold” < =0.01, “hot”/“cold” < = 0.05, “somewhat hot/cold” < = 0.1. Box–Whisker plot depicts the maximum (25th – 1.5 * interquartile range “IQR”) and minimum [75th percentile +1.5 *interquartile range (IQR)], and the Box–Whisker plot depicts median, first (25th percentile) and third (75th percentile) quantiles distribution, each panel represents an ecoregion of Texas (hot-dry, hot-humid, mixed-dry, mixed-humid); For AFL classification, high is >20 ppb, and low ≤20 ppb. The violin plot is shaded in red and depicts the density distribution of the soil property and levels of mycotoxin contamination; and the gray dots depict each data point.

We evaluated the geospatial relationships among the top influential soil properties from the Ratkowsky-ARI nnet model and found commonalities with the nnet models from weather and AFLA-MAIZE ARIs, such as rock fragments (0–25 cm), and calcium carbonate (Figure 8; Supplementary Table S4). The top influential soil features in the Ratkowsky-ARI nnet model were soil rock fragments (0–25 cm), pH (0–50 cm), maximum organic matter (weight fraction), calcium carbonate (kg/m^2^), minimum saturated hydraulic conductivity (μm/s), and soil depth (cm) (Figure 8). Geospatial analysis of rock fragments, pH, maximum organic matter, and calcium carbonate showed a consistent significant hot-spot (higher content than historic statewide average) in the limit regions of hot-dry and hot-humid regions of TX while only rock fragments, pH and calcium carbonate showed a significant cold-spot (lower content than average) in the hot-humid region bordering with Louisiana and the gulf of Mexico (Figure 8). When we analyzed the relationship between AFL contamination levels and the soil properties, we determined that there were negative correlations among high levels of AFL and rock fragments (hot-dry region), maximum organic matter (hot-dry region), calcium carbonate (hot-dry and mixed-dry regions), soil depth (mixed-dry and mixed-humid regions) and a positive correlation with minimum saturated hydraulic conductivity (mixed-dry region) and pH (0–50 cm) (hot-humid area) (Figure 8).

#### 3.3.4 Selection of best models for weather, AFLA-MAIZE and Ratkowsky

To allow predictive modeling for AFL contamination in TX, we compared three models (GBM-standard, GBM-adaboost, and nnet) that were created using weather, AFLA-MAIZE-ARI and Ratkowsky-ARI methods. To evaluate which model and which input features worked best for predicting AFL outbreaks, we used three metrics: accuracy, sensitivity and specificity (Figure 5; Table 2; Supplementary Table S2). Overall, the Ratkowsky-ARI nnet model showed the highest accuracy in single-year validation (73%), highest sensitivity (71%) and greatest specificity (74%). Although weather and AFLA-MAIZE nnet showed higher accuracy, sensitivity and specificity in the test set, compared to Ratkowsky-ARI nnet model, these statistics decreased significantly and fell below Ratkowsky-ARI nnet in the single-year validation analysis (Figure 5; Table 2; Supplementary Table S2).

## 4 Discussion

The integration of ARI in TX included two temperature-dependent growth models: AFLA-MAIZE and Ratkowsky. Both models have been used in agricultural, ecological, and microbiological research to predict how changes in temperature can affect the growth rates of plants, animals, and microorganisms (Ratkowsky and Reddy, 2017; Ratkowsky et al., 1983; Dey et al., 2020). For example, studies on juvenile Arctic cod have shown that temperature-dependent growth models like Ratkowsky can help predict how weather-driven changes in ocean temperatures will impact fish populations (Dey et al., 2020). Similarly, these models have been used to understand the development of pests like the grape berry moth under different temperature conditions (Briere and Pracros, 1998). The AFLA-MAIZE model is based on the beta equation (Battilani et al., 2013), which is flexible and useful in agricultural research contexts that involve different growth phases (Laurel et al., 2017). This model provided a detailed understanding of how temperature fluctuations impact each stage of maize development (Moore et al., 2021). On the other hand, the Ratkowsky model is simpler and highly robust, making it widely applicable across different species and temperature ranges (Ratkowsky and Reddy, 2017; Ratkowsky et al., 1983; Dey et al., 2020). The Ratkowsky equation has been used to model the growth rate of maize under varying temperature conditions, helping to identify the temperature range for optimal growth, which is a crucial consideration for planting schedules and improving yield predictions (Zhu et al., 2021). When comparing beta and Ratkowsky approaches, the beta equation offered detailed fitting capabilities, and the Ratkowsky equation provided a more straightforward approach (with fewer parameters required), making it easier to apply and interpret (Shi et al., 2017; Shi et al., 2016). Overall, both models have their strengths, and the choice between them depends on the specific output requirements and the nature of the data being analyzed (Shi et al., 2017; Shi et al., 2017; Shi et al., 2016).

In the context of mycotoxigenic fungi and prediction of AFL outbreaks, we determined that Ratkowsky-ARI nnet model offered the best performance statistic values when challenged with single-year validation analysis compared to AFLA-MAIZE ARI models (Figure 5; Table 2; Supplementary Table S2). We used both models to predict fungal growth and AFL production in TX and found differences in the range at which the models generate values for ARI. However, this could be linked to the t_min_ and t_max_ constants, which differed between both models by about 1°C. Under the context of pathogenic fungi in the U.S., the AFLA-MAIZE-ARI model was developed using *Aspergillus* section *Flavi* from Italian maize surveys (Battilani et al., 2013; Giorni et al., 2007), while our Ratkowsky-ARI model was developed using AF3357, which was originally isolated from peanuts in the U.S. (Skerker et al., 2021; Hesseltine et al., 1966). This difference in fungal strains could explain the differences in t_min_ and t_max_ from both models. Further studies to refine the Ratkowsky-ARI model would benefit from including a diversity of *A. flavus* genotypes, especially those found at high abundance in U.S. fields.

We evaluated the highly influential input features of our Ratkowsky-ARI nnet model and discovered that ARIs early (March and May), middle (July and August) and late (October and November) every year significantly influenced AFL outbreaks in TX. Because ARI is an engineered feature dependent on temperature, precipitation and relative humidity, the relationship of ARI with fungal growth is dependent on weather variables. It is, perhaps, because of the complex biological feedback loops between fungal growth and the environment that we saw changes in the hot-spots detected for ARIs (Figure 6). *A. flavus* thrives in environments with high humidity and temperature (Mannaa and Kim, 2018), with conditions being above 85% RH and around 30°C (Pratiwi et al., 2015). Furthermore, periods of drought and heat stress can elicit maize physiological stress responses that lead to high AFL contamination under field conditions (Kebede et al., 2012). Therefore, under ideal environmental conditions, the ARI levels are higher and positively correlate with historical AFL levels (Figure 6). Depending on the region and the time of the year, ARI occasionally becomes negatively correlated to AFL levels; this happened in mixed-dry area in July (Week 30) and August (Week 32) and mixed-humid area in August (Week 30). This change in the correlation directionality co-occurred with a switch of the hot-spot changing from hot to cold (mixed-dry area) or cold to hot (mixed-humid area), this phenomenon is likely linked to changes in weather patterns such as temperature and precipitation becoming higher than historic averages in the mixed-dry region in July (Week 30) and August (Week 32) (Figure 7; Supplementary Figure S3). Surveillance of ARI at the beginning and middle of the year could help initiate early and mid-year intervention IPM strategies to minimize biotic and abiotic stresses to the crop, reducing the probability of high AFL concentrations in the grain at harvest time. Though many pre-harvest recommendations for minimizing risk of AFL contamination in Texas such as selection of well-adapted varieties, optimal fertilization, irrigation management (where feasible), and insect control are considered, standard best management practices for maize production (Isakeit, 2011; Pekar et al., 2022), relative risk of AFL outbreaks could be used to prioritize crop management decisions. Risk-based interventions are especially important for regions of Texas where AFL contamination events are perennial and costly management inputs such as application of aflatoxin biocontrol products may not result in a return on investment every year (Outlaw et al., 2016; Wu et al., 2008).

All of our models highlighted the significant role of soil properties in prediction of AFL outbreaks. A common soil property across nnet models from weather, AFLA-MAIZE ARI and Ratkowsky ARI was soil moisture in April (Week 18) and May (Week 20). Microbial communities in the soil are sensitive to soil moisture levels that can support diverse soil organismal communities, enhancing soil health and water retention (Luo et al., 2021). We determined for most of the regions, hot-dry, mixed-humid there was a negative relationship between soil moisture levels and high levels of AFL contamination in week 18 and 20 (Supplementary Figure S4). Interestingly, our findings indicated a positive relationship exists between AFL outbreaks and soil moisture in the mixed-dry region in week 18, meaning that higher soil moisture early in the year was associated with high AFL outbreaks (Supplementary Figure S4). High soil moisture early in the season could cause plants to have a shallower root system (Eapen et al., 2005; Sáenz Rodríguez and Cassab, 2021) and if the field becomes dry later in the season (In the mixed-dry region) then the roots will not be able to reach moisture further down in the soil profile (Sáenz Rodríguez and Cassab, 2021). Thus, the crop will experience increased drought stress and become more susceptible to aflatoxin contamination (Hamidou et al., 2014). It might be advisable to consider the genetic and phenotypic interactions of maize that will be planted in certain regions in TX to select lines with a robust hydrotropic response and higher mesocotyl elongation in response to water scarcity (Sáenz Rodríguez and Cassab, 2021). Our results indicate that there are complex relationships and feedback loops among soil moisture with fungal communities and plant health. It is possible that more diverse soil fungal communities (Frąc et al., 2018) and healthier plants, in high moisture environments, contribute to lower AFL outbreaks (but only in some areas) indicating other confounding factors are important in explaining these contrasting relations. In summary, while certain soils properties such high moisture levels, pH (around 7.0) and high calcium carbonate benefit plant growth, they may also create favorable conditions for pathogenic fungi (Baumgardner, 2012; Liang et al., 2019; Divya et al., 2023). Managing soil health through practices such as crop rotation, proper irrigation, and the use of resistant plant varieties can help mitigate the impacts of these fungi.

Three important soil properties, for prediction of AFL outbreaks from Ratkowsky-ARI nnet model, were soil maximum organic matter, calcium carbonate, and pH (0–50 cm depth). We observed that in the hot-dry region of TX, there was a negative correlation between high levels of AFL and maximum soil organic matter, meaning that higher levels of organic matter in the soil of hot-dry regions tend to have lower AFL levels. Soil organic carbon is a key factor that can modulate the diversity and abundance of fungal pathogens in agricultural soils (Buckman and Brady, 2018). Alteration of organic matter in the topsoil, such as using straw-like mulch in strawberry fields, facilitated an increase in bacterial communities and *Fusarium* derived mycotoxins (Du et al., 2022). The effect of soil organic matter in AFL contamination of maize, however, is yet to be elucidated. We determined that soil calcium carbonate levels were significantly correlated with AFL levels (Figure 4), and high levels of calcium carbonate tend to have lower AFL levels in hot-dry and mixed-dry regions (Figure 8). Also, we found that in TX, there was a positive correlation between AFL levels and soil pH in the hot-dry and hot-humid regions, meaning that the higher the soil pH levels are associated with higher AFL levels (Figures 4, 8). The intersectionality of calcium carbonate and pH levels in the soil with AFL levels has been detected by ML models in Illinois (Castano-Duque et al., 2023). Greater concentrations of dissolved calcium derived from soil parent material with greater CaCO_3_ content led to more alkaline soils and higher soil pH (Weil and Brady, 2002). Direct effects of pH and *A. flavus* growth have shown that the fungus thrives at a pH around 7.5 (Divya et al., 2023) and AFL production increases at more acidic pH levels (Ehrlich, 2014). Soil pH also affects plant health. Maize, however, can grow in soils having a wide pH range (Islam et al., 1980), from 5.0 in the southeast to 8.0 in the western U.S. (Islam et al., 1980; Olson and Sander, 1988), with an optimal pH around 6.5, which also balances plant health with nutrient availability (Olson and Sander, 1988).

The complex interplay between soil pH, plant health and plant-fungus interactions make soil pH a “master soil variable” that influences multi-trophic, chemical and physical processes for plant growth and yield (Neina, 2019). Considering these variables, and geospatial analysis of soil pH hot-spots in TX, the hot-humid region tended to have lower levels of pH compared to the adjacent regions (cold-spot). Overall, soil pH in TX varied from 5.4 to 8.2 and varied largely within counties, as well, which resulted in a range of multi-trophic interactions that positively or negatively affected fungal growth and AFL production, as evidenced here. In the hot-humid areas of TX, IPM strategies targeting soil amendments to achieve optimal pH for plant health, rather than fungal health, could help reduce AFL levels. This recommendation may not apply to other regions of TX where pH levels are already higher than in the hot-humid area. By predicting AFL risk and considering its relationship with soil parameters, heatwaves or cold spells, farmers can implement measures (e.g., adjusting irrigation schedules, selecting heat-tolerant crop varieties (Moore et al., 2021), adding soil amendments, and scheduling time-sensitive biocontrol) to ensure stable and mycotoxin-free yields.

Our selection of the best models developed for predictions of AFL outbreaks considered accuracy, specificity, and sensitivity (Figure 8). Accuracy measured the ratio of correctly predicted high levels of AFL to the total AFL outbreaks (Fox et al., 2017; Parikh et al., 2008). However, accuracy can be misleading in imbalanced datasets where one class dominates (Banerjee et al., 2018), which is the case for AFL outbreaks where the majority of the cases are low contamination events. In our statistics (Table 2; Figure 8; Supplementary Table S2), we observed a significant discrepancy in model performance between the test-set and the validation-set. The training and test-sets were balanced using the SMOTE method (Skerker et al., 2021), while the validation-set was not, challenging the models to predict using an imbalance data set (Single year validation set). For imbalanced datasets, it is crucial to use metrics like precision and sensitivity, instead of only accuracy, to evaluate models (Pratiwi et al., 2015). Given the data imbalance, we evaluated each model’s sensitivity (or recall), which focuses on the ability to correctly identify positive instances (Fox et al., 2017; Banerjee et al., 2018) and specificity, which measured the ability to correctly identify negative instances. This is vital in contexts where false positives are particularly problematic (Fox et al., 2017; Banerjee et al., 2018) such as the case of an AFL outbreak. In terms of specificity, Ratkowsky-ARI nnet model performed better than weather and AFLA-MAIZE-ARI models (Table 2; Figure 8). We attribute this superior performance to the model’s capacity to accurately predict low AFL events, resulting in lower false discovery rate compared to the other two models (Tables 2, 3). These three metrics helped us select a model that not only performed well overall but also aligned with the need to predict rare events (Fox et al., 2017; Banerjee et al., 2018; Montesinos-López and Kismiantini, 2023) like AFL outbreaks.

The application of ARI and weather models was highly dependent on the detection rate, weather, AFLA-MAIZE-ARI and Ratkowsky-ARI models’ detection rates were below 10% (Table 2). To improve detection rate, we performed oversampling techniques such as SMOTE to balance the data-sets and used cross-validation. One of the main constraints in our models is the nature of the training data-set, which lacked robustness. This could improve in future models by conducting a comprehensive survey of mycotoxin contamination events throughout TX that includes all counties that are maize producers. Also, the performance of all the models showed that there is a high biological complexity in TX where there are four major climate zones (Figure 1) that affected the biology and ecology of maize-fungal interactions due to variability in temperature, precipitation, humidity and soil conditions. TX is a prime example where IPM recommendations from our models need to be evaluated under climate region constraints. Our models would suggest that regional specific IPM strategies would be more effective at controlling AFL contamination.

Finally, our implementation of satellite acquired data in the phenology model and ARI calculation risk demonstrates the value and importance of precision agriculture. This approach involves use of geographic information systems (GIS), remote sensing, and predictive modeling to gather detailed information about soil conditions, crop health, and environmental factors (Mona et al., 2018; Giuseppe et al., 2019; Jaime, 2020). These technologies optimize farming practices, enhancing crop yields free of mycotoxins. For example, our predictive models can determine the soil parameters, RH, precipitation and temperature levels that influence AFL outbreaks. This allows farmers to make informed decisions about irrigation, fertilization, and pest control (Mona et al., 2018; Giuseppe et al., 2019; Jaime, 2020). Ultimately, our models strive to incorporate biological complexity by integrating knowledge from multiple disciplines such as agronomy, soil science, mathematics, meteorology and pathology. Further research is needed to model the complex interactions in agriculture at finer spatiotemporal scales due to the dynamic and multifaceted nature of agriculture systems. Our models can support precision agriculture and be instrumental in addressing challenges posed by the environment because there is the potential for simulating variable weather patterns and their effect in AFL outbreaks. This predictive capacity will help farmers adapt by suggesting resilient crop varieties, optimal planting times (Cammarano et al., 2023), better timing for biocontrol application, and soil amendment treatments. Overall, our modeling techniques represent a significant advancement in forecasting aflatoxin contamination while promoting sustainable farming that enables efficient use of resources and better crop management.

## Data Availability

The datasets presented in this study can be found in online repositories. The names of the repository/repositories and accession number(s) can be found at: https://nassgeodata.gmu.edu/CropScape/, https://ladsweb.modaps.eosdis.nasa.gov/missions-and-measurements/products/MCD43A4, https://ldas.gsfc.nasa.gov/nldas/v2/forcing, https://www.nrcs.usda.gov/resources/data-and-reports/soil-survey-geographic-database-ssurgo and https://casoilresource.lawr.ucdavis.edu/soil-properties/.

## Footnotes

^1^ https://www.fda.gov/food/natural-toxins-food/mycotoxins

## Supplementary material

SUPPLEMENTARY FIGURE S1 Ratkowsky fitted curves for **(A)** fungal growth and **(B)** aflatoxin production in relation to variable temperature. Red: fitted values, black: observed values.

SUPPLEMENTARY FIGURE S2 AFLA-MAIZE and Ratkowsky aflatoxin risk index comparison in TX. **(A)** Historic weekly average AFLA-MAIZE ARI by ecoregions from 2003 to 2024. **(B)** Historic weekly average AFLA-MAIZE ARI for Anderson County in year 2008 and 2013. **(C)** Historic weekly average Ratkowsky ARI by ecoregions from 2003 to 2024. **(D)** Historic weekly average Ratkowsky ARI for Anderson County in year 2008 and 2013.

SUPPLEMENTARY FIGURE S3 Geospatial distribution of inputs hot-spots used to engineer top ARI influential features from Ratkowsky-ARI nnet model and their relationship with AFL contamination levels in TX from 2003 to 2024. Average weekly precipitation, relative humidity and temperature in TX during **(A)** week 11 (February); **(B)** week 19 (May); **(C)** week 30 (July); **(D)** week 32 (August); **(E)** week 44 (October); **(F)** week 48 (November). In each panel hotspot geospatial distribution of weather property. Red and blue color palette of geospatial hot-spot analysis used the historic mean of gi-value for weekly average precipitation as the middle point scale, red hues are gi-values above the historic mean, and blue hues are below the historic mean. Hot-spot specific red/blue hues are classified by the level of significance of the p-folded value: “very hot/cold” <= 0.01, “hot”/“cold” <= 0.05, “somewhat hot/cold” <= 0.1. Box–Whisker plot depicts the maximum (25th – 1.5 * interquartile range “IQR”) and minimum [75th percentile + 1.5 *interquartile range (IQR)], and the Box–Whisker plot depicts median, first (25th percentile) and third (75th percentile) quantiles distribution, each panel represents an ecoregion of Texas (Hot-dry, hot-humid, mixed-dry, mixed-humid); For AFL classification, high is >20 ppb, and low ≤20 ppb. The violin plot is shaded in red and depicts the density distribution of weekly average precipitation and levels of mycotoxin contamination; and the gray dots depict each data point.

SUPPLEMENTARY FIGURE S4 Geospatial distribution of top influential soil moisture features in the Ratkowsky ARI nnet model in TX from 2003 to 2024. **(A)** Hot-spots for ARI in week 4 (January), **(B)** Average weekly precipitation in week 4, **(C)** average weekly temperature in week 4, **(D)** average weekly relative humidity in week 4, **(E)** hot-spots for ARI in week 28 (July), **(F)** Average weekly precipitation in week 28, **(G)** average weekly temperature in week 28, and **(H)** average weekly relative humidity in week 28. Maps of geospatial distribution of the weekly ARI are shaded in red from 2003 to 2021 for each specific week, the y-axis is latitude, and the x-axis is longitude. Red and blue color palette of geospatial hot-spot analysis used the historic mean of gi-value for weekly ARI as the middle point scale, red hues are gi-values above the historic mean, and blue hues are below the historic mean. Hot-spot specific red/blue hues are classified by the level of significance of the p-folded value: “very hot/cold” <= 0.01, “hot”/“cold” <= 0.05, “somewhat hot/cold” <= 0.1.

SUPPLEMENTARY Table 1 Accuracies of all AFL models generated using all-weather, AFLA-MAIZE or Ratkowksy ARI from test-set and single year-set by using GBM-standard, GBM-adaboost, nnet and dnn. *Weather parameters were temperature, precipitation and relative humidity, these parameters were used to feature engineer ARI. Test-set used was 30% of the data and single year validation-set was 2013.

SUPPLEMENTARY Table 2 Summary statistics of all AFL models generated using all-weather, AFLA-MAIZE or Ratkowksy ARI from test-set and single year-set by using GBM-standard, GBM-adaboost, nnet and dnn. *Weather parameters were temperature, precipitation and relative humidity, these parameters were used to feature engineer ARI. Test-set used was 30% of the data and single year validation-set was 2013.

SUPPLEMENTARY Table 3 Contingency tables of all AFL models generated using all-weather, AFLA-MAIZE or Ratkowksy ARI from test-set and single year-set by using GBM-standard, GBM-adaboost, nnet and dnn. *Weather parameters were temperature, precipitation and relative humidity, these parameters were used to feature engineer ARI. Test-set used was 30% of the data and single year validation-set was 2013.

SUPPLEMENTARY Table 4 Summary of input features importance of all AFL models generated using all-weather, AFLA-MAIZE or Ratkowksy ARI from test-set and single year-set by using GBM-standard, GBM-adaboost, nnet and dnn. *Weather parameters were temperature, precipitation and relative humidity, these parameters were used to feature engineer ARI. Test-set used was 30% of the data and single year validation-set was 2013.
