# Supplementary figures and images for: Prediction of aflatoxin contamination outbreaks in Texas corn using mechanistic and machine learning models
Source: Front Microbiol. 2025 Mar 5;16:1528997. doi: 10.3389/fmicb.2025.1528997 (PMC11919900; doi:10.3389/fmicb.2025.1528997)

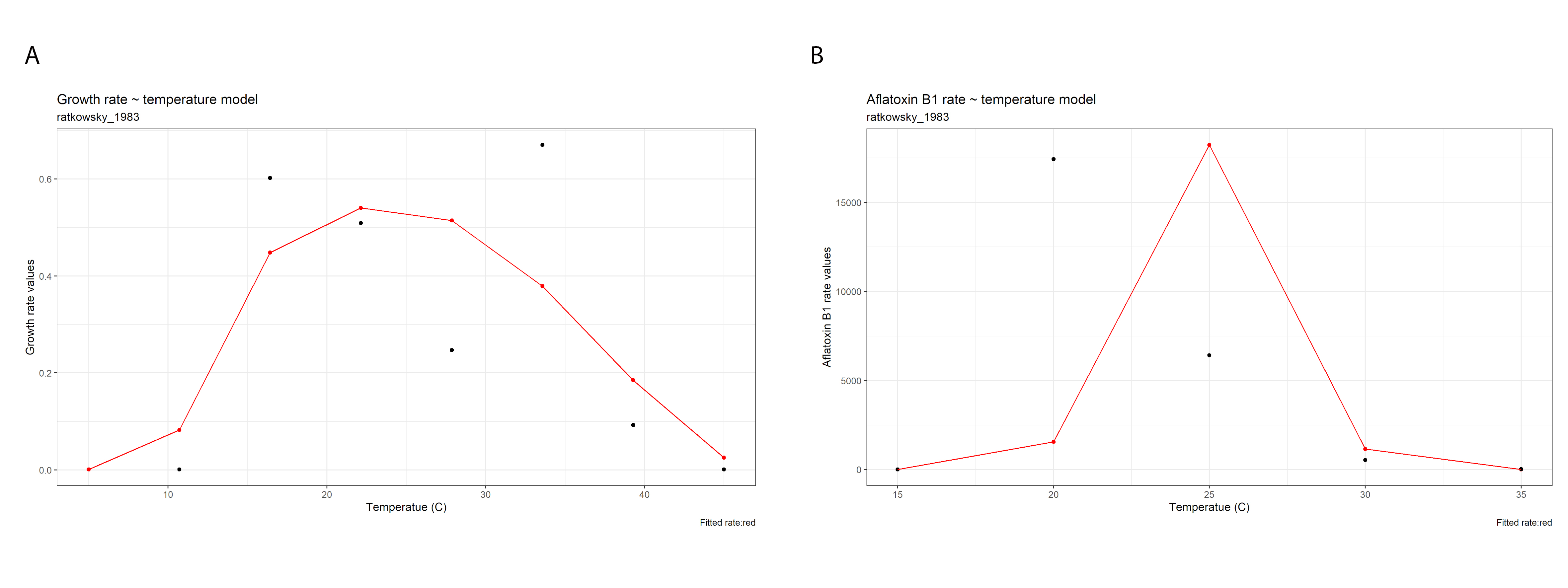

Supplement: SUPPLEMENTARY FIGURE S1 — Ratkowsky fitted curves for (A) fungal growth and (B) aflatoxin production in relation to variable temperature. Red: fitted values, black: observed values. [file Image_1.jpeg]

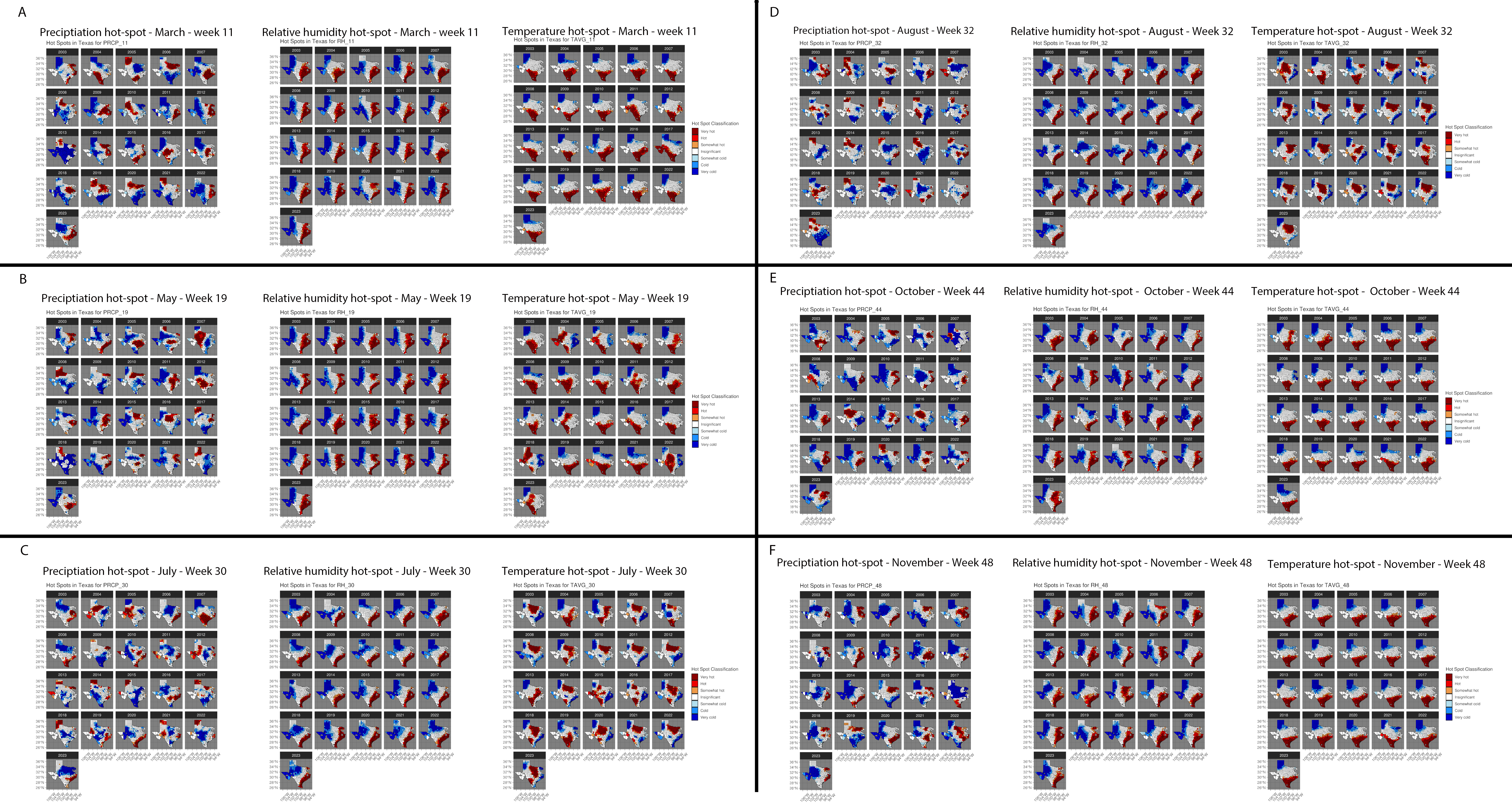

Supplement: SUPPLEMENTARY FIGURE S3 — Geospatial distribution of inputs hot-spots used to engineer top ARI influential features from Ratkowsky-ARI nnet model and their relationship with AFL contamination levels in TX from 2003 to 2024. Average weekly precipitation, relative humidity and temperature in TX during (A) week 11 (February); (B) week 19 (May); (C) week 30 (July); (D) week 32 (August); (E) week 44 (October); (F) week 48 (November). In each panel hotspot geospatial distribution of weather property. Red and blue color palette of geospatial hot-spot analysis used the historic mean of gi-value for weekly average precipitation as the middle point scale, red hues are gi-values above the historic mean, and blue hues are below the historic mean. Hot-spot specific red/blue hues are classified by the level of significance of the p-folded value: “very hot/cold” <= 0.01, “hot”/“cold” <= 0.05, “somewhat hot/cold” <= 0.1. Box–Whisker plot depicts the maximum (25th – 1.5 * interquartile range “IQR”) and minimum [75th percentile + 1.5 *interquartile range (IQR)], and the Box–Whisker plot depicts median, first (25th percentile) and third (75th percentile) quantiles distribution, each panel represents an ecoregion of Texas (Hot-dry, hot-humid, mixed-dry, mixed-humid); For AFL classification, high is >20 ppb, and low ≤20 ppb. The violin plot is shaded in red and depicts the density distribution of weekly average precipitation and levels of mycotoxin contamination; and the gray dots depict each data point. [file Image_3.jpeg]

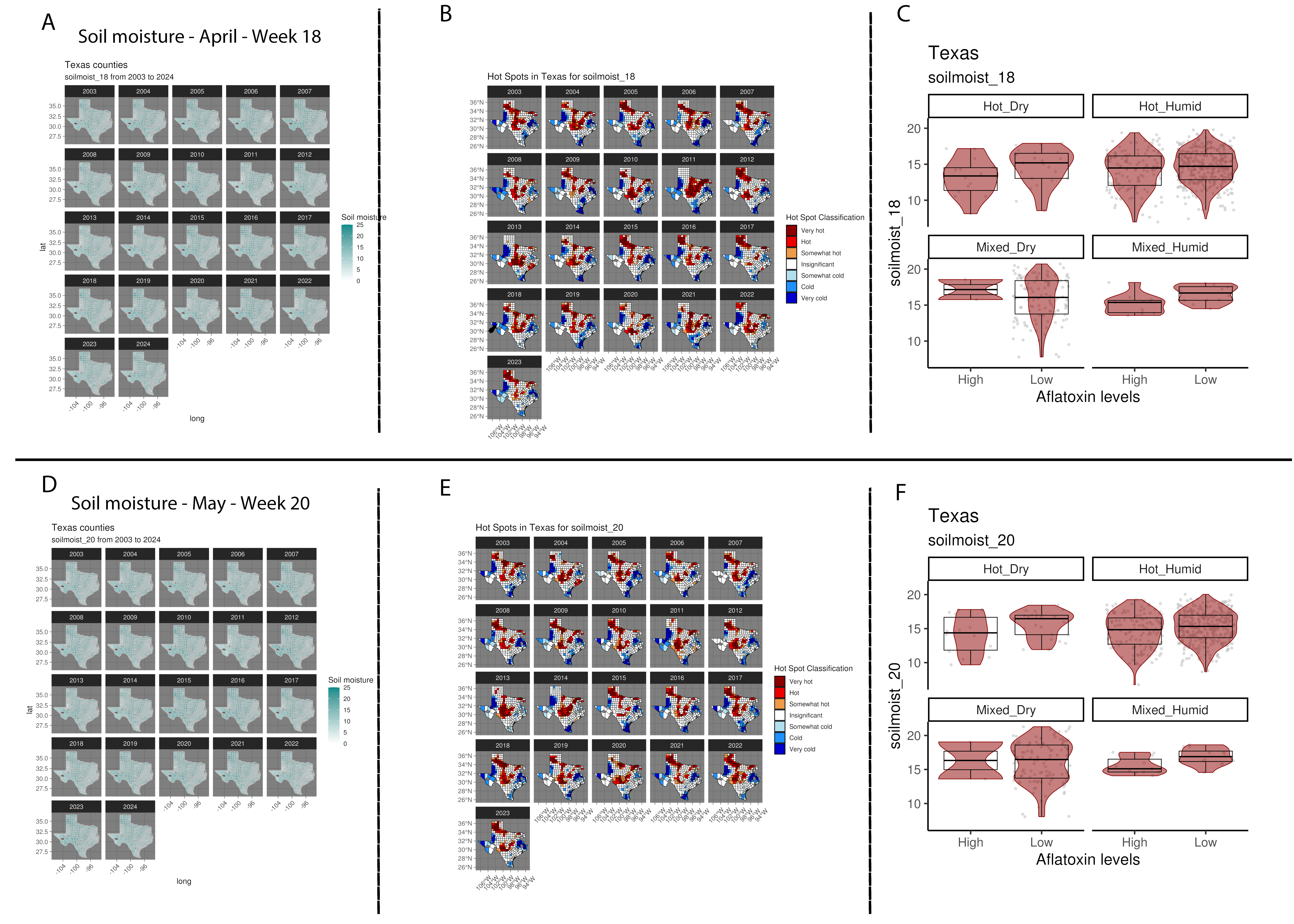

Supplement: SUPPLEMENTARY FIGURE S4 — Geospatial distribution of top influential soil moisture features in the Ratkowsky ARI nnet model in TX from 2003 to 2024. (A) Hot-spots for ARI in week 4 (January), (B) Average weekly precipitation in week 4, (C) average weekly temperature in week 4, (D) average weekly relative humidity in week 4, (E) hot-spots for ARI in week 28 (July), (F) Average weekly precipitation in week 28, (G) average weekly temperature in week 28, and (H) average weekly relative humidity in week 28. Maps of geospatial distribution of the weekly ARI are shaded in red from 2003 to 2021 for each specific week, the y-axis is latitude, and the x-axis is longitude. Red and blue color palette of geospatial hot-spot analysis used the historic mean of gi-value for weekly ARI as the middle point scale, red hues are gi-values above the historic mean, and blue hues are below the historic mean. Hot-spot specific red/blue hues are classified by the level of significance of the p-folded value: “very hot/cold” <= 0.01, “hot”/“cold” <= 0.05, “somewhat hot/cold” <= 0.1. [file Image_4.jpeg]
